# Optimizing management of uncomplicated acute malnutrition in children in rural Niger: a 3-arm individually randomized, controlled, noninferiority trial

**DOI:** 10.1016/j.ajcnut.2025.07.004

**Published:** 2025-09-01

**Authors:** Maguy Daures, Jérémie Hien, Kevin Phelan, Harouna Boubacar, Sanoussi Atte, Mahamadou Aboubacar, Ahmad AGM Aly, Baweye Mayoum, Jean-Claude Azani, Jean-Jacques Koffi, Benjamin Seri, Aurélie Beuscart, Valérie Journot, Victoire Hubert, Moumouni Kinda, Xavier Anglaret, Cécile Cazes, Suvi Kangas, André Briend, Susan Shepherd, Renaud Becquet

**Affiliations:** 1 National Institute for Health and Medical Research (Inserm) UMR 1219, Research Institute for Sustainable Development (IRD) EMR 271, Bordeaux Population Health Research Centre, University of Bordeaux, Bordeaux, France; 2 The Alliance for International Medical Action (ALIMA), Zinder, Niger; 3 The Alliance for International Medical Action (ALIMA), Paris, France; 4 Nutrition Directorate, Ministry of Health, Niamey, Niger; 5 Commission for the Initiative “les Nigériens Nourrissent les Nigériens” (HC3N), Niamey, Niger; 6 The Alliance for International Medical Action (ALIMA), Dakar, Senegal; 7 PACCI Research Programme, University Hospital of Treichville, Abidjan, Côte d'Ivoire; 8 International Rescue Committee, Brussels, Belgium; 9 University of Copenhagen, Frederiksberg, Denmark; 10 Tampere Center for Child, Adolescent and Maternal Health Research, Faculty of Medicine and Health Technology, Tampere University, Tampere University Hospital, Tampere, Finland

**Keywords:** acute malnutrition, children, simplified protocol, mid-upper arm circumference, clinical trial, sub-Saharan Africa

## Abstract

**Background:**

The Optimizing treatment for acute MAlnutrition (OptiMA) and Combined Protocol for Acute Malnutrition Study (ComPAS) strategies aim to simplify acute malnutrition treatment programs by treating severe and moderate cases with one product—ready-to-use therapeutic food (RUTF)—at gradually reduced doses.

**Objectives:**

This study aims to evaluate the noninferiority of treatment under OptiMA and ComPAS compared with Niger’s standard protocol in children aged 6–59 mo included with *1*) mid-upper arm circumference (MUAC) <125 mm or edema who achieved the primary outcome, defined as being alive, not acutely malnourished according to the inclusion criteria (MUAC ≥ 125 mm, weight-for-height z-score ≥−3, and no edema) and not having experienced an additional episode of acute malnutrition during a 6-mo period; *2*) children with MUAC < 115 mm or edema who achieved nutritional recovery, defined as MUAC ≥ 125 mm and no edema for 2 wk; temperature <37.5°C; 4-wk minimum treatment.

**Methods:**

This individually randomized controlled trial (NCT04698070) was conducted in Niger 2021 to 2022. Children were assigned to either Standard, OptiMA, or ComPAS. Noninferiority, using 10% margin, was assessed in intention-to-treat (ITT) and per-protocol (PP) analyses.

**Results:**

For the primary outcome among 1732 children with acute malnutrition, noninferiority was demonstrated for ComPAS {51.9%, difference 1.0% [97.5% confidence interval (CI): –5.5%, +7.6%]} and OptiMA [49.7%, difference 3.2% (–3.3%, +9.9%)] in ITT analysis, but only for ComPAS in PP analysis. Among 1140 children with MUAC <115 mm or edema, standard protocol was superior in terms of nutritional recovery in ITT and PP analysis. There was no difference in mortality across the arms over 6 mo, and growth trajectories were similar between Standard and ComPAS, which used 50% less RUTF, but lower in OptiMA which used 32% less RUTF. A post-hoc analysis showed noninferiority of both intervention arms when 1-wk recovery was the defined endpoint, and 20% more missed visits in OptiMA and ComPAS.

**Conclusions:**

Only the ComPAS protocol demonstrated noninferiority to standard treatment in ITT and PP analyses for children with MUAC <125 mm or edema. The OptiMA protocol’s lower performance is unclear, but it suggests that reduced RUTF dosage had limited influence on overall child growth, as ComPAS used less RUTF than OptiMA. For the most malnourished children with MUAC <115 mm or edema, the standard protocol showed a higher recovery proportion, primarily due to better attendance rather than differences in mortality or growth at 6 mo. Further studies are needed to better understand the acceptability and population-level impact of simplified protocols in multiple settings.

## Introduction

Acute malnutrition is a major public health problem, with an estimated 45 million children aged under 5 y in 2022, including 13.6 million suffering from severe acute malnutrition (SAM), based solely on weight-for-height *z*-scores (WHZ) [1]. Acute malnutrition is also a leading underlying cause of mortality in children under 5, contributing to ≥800,000 deaths annually worldwide [2]. Although the data are alarming, these prevalences underestimate the true burden, as they do not account for the annual incidence of acute malnutrition or children identified by mid-upper arm circumference (MUAC) or nutritional edema [[1], [2], [3]].

Community-based management of acute malnutrition programs, which have become the cornerstone of global acute malnutrition treatment, face chronic underfunding and significant gaps in coverage. Less than 25% of children with SAM are admitted for treatment [4] and the costs of these programs are largely driven by the use of ready-to-use therapeutic food (RUTF) and human resources [5]. Paradoxically, in standard protocols, children receive more RUTF as they approach recovery rather than during the critical, life-threatening phase of treatment when weight gain is most rapid [6,7]. Evidence suggests that this approach may not provide additional growth benefits during the latter stages of recovery [7].

Acute malnutrition treatment is further complicated by its division into separate protocols for moderate and severe cases and a complex definition. Moderate acute malnutrition is defined by the WHO as a MUAC between 115 and 124 mm or a WHZ between –2 and –3, whereas SAM is defined by a MUAC <115 mm, WHZ <–3, or the presence of bilateral edema [8]. These programs are managed separately, supported by different United Nations agencies, and use different protocols and products [ready-to-use supplementary food (RUSF) or fortified-blended flours for moderate acute malnutrition; RUTF for SAM]. Such separation complicates delivery of care, contributes to low coverage, and creates confusion among caregivers [9].

In response to these challenges, several approaches to simplify processes and increase treatment access among children aged 6–59 mo have been tested in sub-Saharan Africa [[10], [11], [12], [13], [14], [15]]. Protocol modifications are varied and still evolving but common threads include: *1*) using only MUAC or edema for screening, admission, follow-up and discharge, *2*) a combined treatment of all children with acute malnutrition defined as MUAC <125 mm or presence of bipedal edema, *3*) a single product for treatment (RUTF) at progressively reduced dosages adapted to the severity of acute malnutrition at admission and during treatment. In line with these studies, the WHO updated in June 2023 their malnutrition treatment guidelines to include the use of RUTF for treating moderate acute malnutrition as well as reduced dosage of RUTF in some contexts for children admitted with SAM once they no longer have severe criteria [8].

Among these approaches, the Optimizing treatment for acute MAlnutrition (OptiMA) strategy calibrates the RUTF ration to the child’s degree of wasting based on the combination of MUAC status and weight whereas the Combined Protocol for Acute malnutrition Study (ComPAS) determines RUTF ration based on MUAC alone and provides less RUTF than OptiMA. Initial trials in Central and East Africa have demonstrated the effectiveness of these simplified approaches including dosage reduction [13,15,16]. The latest WHO recommendations now call for further studies to compare different protocol options with reduced dosages, both against each other and standard dosages. Additionally, because the determinants of acute malnutrition are multifactorial and context-dependent [17], further evidence is needed in the Sahel region, particularly in countries like Niger, which bears a high burden of malnutrition. Finally, it is particularly important to understand the impact of reduced RUTF regimens on nutritional recovery among the most severely wasted children. To date, no trial has directly tested RUTF dose reduction in children admitted with MUAC <115 mm or edema, a group that has been shown to have lower recovery and higher treatment nonresponse proportions [14,18].

We are reporting here the results of a 3-arm noninferiority individually randomized trial in rural Niger, comparing the OptiMA and ComPAS strategies to the current Nigerien national protocol for the treatment of acute malnutrition in children [19], with primary and priority secondary outcomes evaluated over 6 mo among children with MUAC <125 mm or edema and those with MUAC <115 mm or edema.

## Methods

### Study design

This 3-arm open-label, community-based, individually randomized, controlled noninferiority trial was conducted in 4 health zones (Diney, Droum, Gada, and Gafati) of Mirriah Health District, Zinder Region, in Niger. The Alliance for Medical Action (ALIMA) and its partner local nongovernmental organization in Niger, Bien-être de la Femme et de l’Enfant, have supported the Ministry of Health in Mirriah district since 2009 to provide medical services to women and children aged under 5 y. The population in Mirriah District was estimated at 700,000 inhabitants in 2019, with the combined population of the 4 health areas in this trial being 178,535. A survey using the Standardized Monitoring and Assessment of Relief and Transitions approach conducted in 2020 showed high prevalence of global acute malnutrition defined by MUAC <125 mm or edema of 10.5% [95% confidence interval (CI): 9.3%, 11.9%] and SAM defined by MUAC < 115 mm or edema of 3.2% (95% CI: 2.6%, 3.9%) in Zinder region [20]. A full description of this trial protocol was previously published [21].

Ethical approval was given from the National Ethics Committee in Niger (approval number: 065/2020/CNERS) and from the Ethics Evaluation Committee of the French National Institute for Health and Medical Research (INSERM) in France (approval number: 21-025). Children were enrolled in the study after written informed consent had been given by their caregivers. Care was provided free of charge, regardless of participation in the study. The trial is registered on clinicaltrials.gov (NCT04698070).

### Participants

Children were eligible for inclusion if they were aged 6–59 mo, presenting for outpatient consultation at any of the 4 primary health centers without medical complications and with ≥1 of the 3 following acute malnutrition criteria: MUAC <125 mm and/or bilateral pitting edema (feet or ankles: +; feet or lower legs and hands or lower arms: ++) and/or WHZ <−3.

There were 2 groups of children in this study. First, children who were included but not randomly assigned: siblings of already enrolled children or those with WHZ <−3 and MUAC ≥125 mm who were automatically allocated to the standard arm as they were eligible for treatment with the National protocol but not in either intervention arms. Second, children who were eligible for randomization, those with MUAC <125 mm and/or bilateral edema, were randomly assigned to 1 of the 3 study arms after informed consent was obtained.

### Randomization and masking

Randomization was stratified by severity of acute malnutrition (MUAC <115 mm or edema, MUAC ≥115 mm and WHZ <–3, MUAC 115–124 mm and WHZ ≥–3) to ensure reaching the required sample size for primary objectives, before allocation to 1 of the 3 treatment strategies. Children with MUAC < 125 mm and/or bilateral edema were randomly assigned (1:1:1) to either the OptiMA or ComPAS arms (intervention = OptiMA or ComPAS) or the national standard protocol arm (control = Standard). Randomization was performed using specially developed software uploaded onto tablets that hosted the randomization lists prepared in advance by an independent statistician and which were inaccessible to trial staff. After verifying eligibility, the clinical trial officer ran the randomization software which assigned a code and corresponding treatment arm. Confidential randomization blocks were used, stratified by study site and MUAC < 115 mm or edema. Due to evident differences between RUTF ration, clinic staff and caregivers could not be blinded.

### Interventions

Comparison of OptiMA, ComPAS, and standard protocol dosage tables is detailed in Supplemental Tables 1 and 2.

In the standard arm, children included with a MUAC < 115 mm and/or WHZ <–3 and/or bilateral edema were treated weekly with RUTF at 150–200 kcal/kg/d during the entire treatment schedule until discharge defined as reaching a MUAC > 125 mm and WHZ >–2 z-score for 2 consecutive weeks without edema, according to the Niger national protocol. Children aged under 2 y included with a MUAC between 115 and 124 mm and WHZ >–3 without bipedal edema were treated weekly by 1 sachet of RUSF per day (500 kcal/d) during the course of treatment. Children aged over 2 y meeting the same moderate anthropometric criteria were not eligible for supplementation according to the national moderate acute malnutrition program.

All children aged 6–59 mo who met eligibility criteria received therapeutic food in the intervention arms. In the OptiMA strategy, children with MUAC < 115 mm received 170–200 kcal/kg/d of RUTF, children with MUAC 115–119 mm, either at admission or during the course of treatment, received 125–190 kcal/kg/d of RUTF, and children with MUAC 120–124 mm received 50–166 kcal/kg/d of RUTF (with a minimum of 1 sachet/d) until discharge. The ComPAS strategy provided RUTF according to the child’s MUAC category at each clinic visit, irrespective of weight: 2 sachets/d of RUTF (1000 kcal/d) to children with MUAC < 115 mm or edema and 1 sachet/d (500 kcal/d) for children with MUAC between 115 and 124 mm. Children with edema received the same RUTF ration as children with MUAC < 115 mm until edema was resolved, and then followed the dosage of their allocated arms.

### Monitoring follow-up

All participants were monitored for 6 mo post randomization. Children eligible for supplementation had weekly health center appointments until they reached the discharge criteria of their allocated arm (Supplemental Table 1). On discharge from treatment, or immediately after inclusion for moderate acute malnutrition children aged over 2 y in standard arm (not eligible to supplementation), children were seen once a month at home by a nurse research officer assisted by 1 or 2 community health workers (CHWs) until the 6-mo study period was completed.

Children who failed to reach recovery criteria after 16 wk of supplementation were classified as nonresponders to therapeutic feeding and were transferred from weekly clinic visits to monthly home visits. Nonresponders with MUAC persistently <115 mm after 14–16 wk of therapeutic feeding were reviewed at weekly medical meetings to ascertain that appropriate measures had been taken for additional investigations. Every effort was taken to test these children for HIV, anemia, and tuberculosis. Children who did not come to the planned visits at the health center were traced by CHWs at home. If the child was not seen at home and no vital status was known, he was notified as lost to follow-up.

At all health centers or home visits, vital signs, anthropometric measures, and clinical characteristics were collected. Additional data were collected at health center visits on RUTF/RUSF supplementation and amoxicillin or other treatment prescribed. To ensure mothers’ adherence, 6 and 8 soaps were given to caregivers at inclusion, at the end of outpatient follow-up and at each home visit in standard and intervention arms, respectively.

All other aspects of the standard nutrition protocol, such as systemic medications, hospitalization, were similarly applied to all children in the 3 arms following the national recommendations [19].

### Outcomes

Among children with MUAC < 125 mm or edema, the primary outcome was a composite variable of being alive, not acutely malnourished according to the inclusion criteria (MUAC ≥ 125 mm, WHZ ≥ −3, and no edema) and not having experienced an additional episode of acute malnutrition during a 6-mo observation period. Children who died, were lost to follow-up, failed to recover as defined above or moved out of the study area with their families during the study period, and those who had a new episode of acute malnutrition within the period (that is, relapse) were considered as unsuccessful. Relapse was defined, after the child had previously met the criteria for the primary outcome (that is, MUAC ≥ 125 mm and WHZ score ≥−3 without edema), as a drop in one of these criteria below the recovery thresholds until at least a 6-mo follow-up was reached.

The main secondary outcome was nutritional recovery and was measured among children with MUAC < 115 mm or edema at inclusion. This outcome was defined in each arm by a 4-wk minimum duration of treatment, an axillary temperature <37.5°C, and an absence of bipedal edema and a MUAC > 125 mm for 2 consecutive week, and could be reached at any point during the 6-mo follow-up period, regardless of the discharge status at health center.

Other secondary outcomes were:

1. Nutritional and anthropometric characteristics over 6 mo post inclusion such as MUAC, WHZ, WAZ, and HAZ at 6 mo, as well as changes in weight and MUAC from inclusion to 6 mo.
2. Nutritional improvement at different time points, including recovery at 12 and 16 wk. An alternative definition of recovery (post-hoc analysis) was considered, based on achieving once a MUAC ≥ 125 mm and no bilateral nutritional edema at any point during the 6-mo follow-up period.
3. The total amount of RUTF/RUSF received over 6 mo and the time to nutritional recovery were also analyzed.
4. Follow-up characteristics during the 6-mo trial period including program attendance, incidence of comorbidities, and hospitalizations.

### Sample size

Our sample size was designed to detect noninferiority of an expected 68% of the study population achieving the primary outcome in each arm and anticipating 74% of children with MUAC < 115 mm or edema achieving the main secondary objective. We assumed a noninferiority margin of 10%, as generally recommended per the European Medicines Agency and the United States Food and Drug Administration guidelines, a one-sided type I error of 1.25, a power of 90% for the primary outcome and 80% for the main secondary outcome. In this trial, with an expected recovery proportion of 68%, a 10% noninferiority margin was considered both acceptable and conservative, aligning with margins used in comparable studies such as the ComPAS trial, the OptiMA-Democratic Republic of Congo (DRC) trial, and the Mango trial [12,13,15]. An inflation rate of 5% was applied to account for unusable data. For the primary objective, 567 children per arm (*N* = 1701) were required and 384 children (*N* = 1152) for the main secondary objective.

Recruitment occurred in 2 phases to ensure adequate statistical power for measuring the 2 main outcomes. In the first phase, all children with MUAC < 125 mm or edema were eligible and randomly assigned. In the second phase, only children with MUAC < 115 mm or edema (+, ++) continued to be randomly assigned. This continued until the combined number of children with MUAC < 115 mm or edema enrolled during the first and second phases reached the sample size required for the main secondary outcome analysis.

### Statistical analysis

All the analyses are presented in the 2 populations of children, including those with MUAC < 125 mm or edema, and those with MUAC < 115 mm or edema. Qualitative variables were described in terms of numbers and percentages and compared using Chi-square or Fisher's exact tests. Quantitative variables were described using means and SD or medians and IQR, with comparisons made using Student’s *t*-test, Wilcoxon’s or Kruskal-Wallis test, depending on the distribution of the variable of interest. A *P* value < 0.05 between the standard and intervention arms was considered statistically significant. For the noninferiority test, the CI was calculated using the “diffscoreci” function from the PropCIs package of the R software, estimating the score interval for the difference in proportions between independent samples.

Noninferiority analyses were planned to compare the primary objective and the main secondary objective in the intention-to-treat (ITT) and per-protocol (PP) populations. ITT analysis included all participants. PP analysis included participants who had a minimum prescription of 4 wk’ RUTF, received ≥90% of the total amount of RUTF/RUSF they were supposed to receive as per the protocol (Supplemental Table 2), and had a maximum interval of 8 wk (one home visit missed) between any 2 visits over the study period. The OptiMA or ComPAS strategies were considered noninferior to the standard strategy if the upper bound of the 97.5% CI for the difference between standard and OptiMA or ComPAS groups was lower than 10%, both in the ITT and PP analyses, as recommended by CONSORT [22]. We designed this trial using ITT as the main analysis, as it better assesses the mean effect of assigning a drug to a group of patients [23].

In post-hoc analysis, we used mixed-effect generalized additive models adjusted on age, sex, or WHZ at admission for fitting and plotting means of anthropometric parameters, such as weight, MUAC, and height. We represented a 95% CI band around each curve to compare randomization groups.

Duplicate analyses were performed for the main outcomes by the scientific project manager (MD) and a senior scientist from the INSERM team (VJ) using 2 statistical software, R version 4.1.2 and SAS version 9.4, respectively.

### Role of the funding source

The funders of the study had no role in study design, data collection, data analysis, data interpretation, or writing of the report. All authors had full access to all the data in the study. RB and SS had final responsibility for the decision to submit the manuscript for publication.

## Results

**FIGURE 1:**
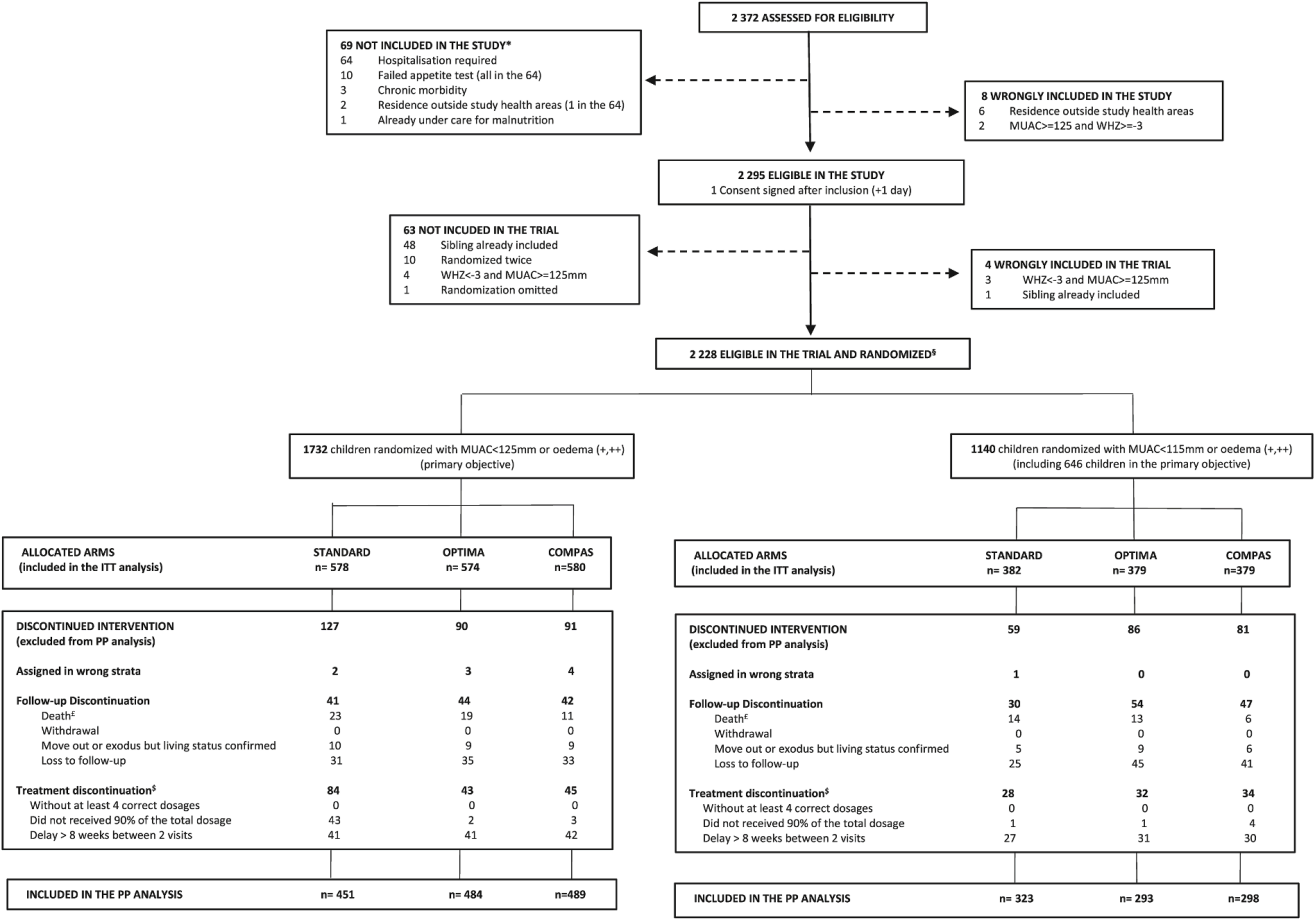
Trial profile. ^§^Recruitment was conducted in 2 phases: in phase 1, all children with MUAC < 125 mm or edema were randomized; in phase 2, only children with MUAC < 115 mm or edema were randomly assigned until the target sample size for the main secondary outcome was reached. Two children were randomly assigned among SAM children with MUAC < 115 or WHZ < –3 or edema (+, ++) after reaching the sample size needed for children with MUAC < 125 mm so they are only included in the analysis of children with SAM presented in Supplementary Tables 3–6 and Supplementary Figure 1. ITT analysis included all participants. Per-protocol definition: minimum prescription of 4 wk of supplementation, children received ≥90% of the total amount of supplementation they were supposed to receive as per protocol and a maximum interval of 8 wk between any 2 visits in the 6-mo follow-up. ^£^Not excluded from PP analyses. ^$^Among children who completed the 6-mo follow-up. Number of children expected in ITT analyses: 568 for the primary objective; 384 in secondary priority objective. ComPAS, Combined Protocol for Acute Malnutrition Study; ITT, intention-to-treat analysis; MUAC, mid-upper arm circumference; OptiMA, Optimizing treatment for acute MAlnutrition; PP, per protocol analysis; SAM, severe acute malnutrition; WHZ, weight for height z-score.

The flowchart of the OptiMA trial is shown in Figure 1. During the first phase, between March 31 and September 21, 2021, we randomly assigned 1732 children with MUAC < 125 mm or edema including 578, 574, and 580 in standard, OptiMA, and ComPAS arms in ITT analysis, respectively. For the PP analysis, 127 (22.0%) children were excluded in the standard arm compared with 90 (15.7%) in OptiMA and 91 (15.7%) in ComPAS. This difference was mainly explained by the higher number of children who did not receive 90% of the total dosage (that is, dosage error) in the standard arm (43 children in standard compared with 2 in OptiMA, and 3 in ComPAS). During the second phase until 23 December, 2021, we continued to enroll children until the total number of children with MUAC < 115 mm or edema (+, ++) at admission (recruited during both the first and second phases) reached the sample size required for the main secondary outcome. In total, 1140 children with MUAC < 115 mm or edema (+, ++) at baseline were enrolled, including 382, 379, and 379 in standard, OptiMA, and ComPAS arms in ITT analysis, respectively. For the PP analysis, 59 (15.4%) were excluded in the standard arm compared with 86 (22.7%) in OptiMA and 81 (21.4%) in ComPAS. The difference was mainly explained by the higher number of lost-to-follow-up children in OptiMA (*n* = 45) and ComPAS (*n* = 41) compared with standard (*n* = 25).

Initially, this trial also aimed to assess the noninferiority of the simplified strategies in children with SAM according to the WHO definition (MUAC < 115 mm or WHZ <–3 or edema). Among these children, the majority (651, or 73%) also had MUAC < 115 mm or edema, resulting in similar outcomes between the 2 groups. The results for children with SAM according to the WHO definition are presented in Supplemental Tables 3–6 and Supplemental Figure 1.

### Results for children randomly assigned with MUAC < 125 mm or edema (+, ++)

Baseline sociodemographic, anthropometric, and clinical characteristics are presented in Table 1. A 6% difference was observed in the sex distribution; more girls were enrolled in the intervention arms. We also observed 94 (16.3%), 93 (16.2%), and 127 (21.9%) children admitted with WHZ ≥–2 in the standard, OptiMA, and ComPAS arms, respectively. All other characteristics were similar in the 3 arms. The overall median age was 15 mo (IQR: 10–22) and the median MUAC was 116 mm (IQR: 112–120). In the standard arm, 42 (7.3%) moderate acute malnutrition children were not eligible for supplementation at admission because they were aged over 24 mo whereas all children in the intervention arms started RUTF.

**TABLE 1:** Baseline characteristics

Variable	Children with MUAC < 125 mm or edema (+, ++)			Children with MUAC < 115 mm or edema (+, ++)		
	Standard (*N* = 578)	OptiMA (*N* = 574)	ComPAS (*N* = 580)	Standard (*N* = 382)	OptiMA (*N* = 379)	ComPAS (*N* = 379)
Sociodemographic characteristics						
Female gender	279 (48.3)	314 (54.7)	320 (55.2)	208 (54.5)	219 (57.8)	217 (57.3)
Age (mo)	15 (10, 22)	15 (10, 22)	15 (10, 21)	14 (9, 22)	14 (9, 22)	14 (9, 22)
Health center						
Diney	84 (14.5)	83 (14.5)	85 (14.7)	33 (8.6)	33 (8.7)	33 (8.7)
Droum	211 (36.5)	208 (36.2)	211 (36.4)	190 (49.7)	185 (48.8)	188 (49.6)
Gada	112 (19.4)	112 (19.5)	113 (19.5)	72 (18.8)	73 (19.3)	72 (19.0)
Gafati	171 (29.6)	171 (29.8)	171 (29.5)	87 (22.8)	88 (23.2)	86 (22.7)
Currently breastfed	334 (57.8)	347 (60.5)	365 (62.9)	224 (58.6)	241 (63.6)	232 (61.2)
Mother alive	575 (99.5)	574 (100.0)	573 (98.8)	380 (99.5)	378 (99.7)	374 (98.7)
Mother as caretaker	540 (93.4)	551 (96.0)	556 (95.9)	355 (92.9)	366 (96.6)	361 (95.3)
Caretaker was illiterate	532 (92.0)	533 (92.9)	529 (91.2)	371 (97.1)	370 (97.6)	365 (96.3)
First child born	30 (5.2)	16 (2.8)	27 (4.7)	14 (3.7)	9 (2.4)	10 (2.6)
Distance from health center ≥10 km	126 (21.8)	120 (20.9)	131 (22.6)	125 (32.7)	109 (28.8)	130 (34.3)
Number of siblings	4·0 (2.0, 6.0)	4.0 (2.0, 6.0)	4.0 (2.0, 6.0)	4.0 (2.0, 6.0)	4.0 (2.0, 6.0)	4.0 (2.0, 5.0)
Number of people living in the household	6·0 (4.0, 9.0)	6.0 (4.0, 9.0)	6.0 (5.0, 9.0)	6.0 (4.0, 9.0)	6.0 (4.0, 9.0)	6.0 (5.0, 9.0)
More than one child participating in OTP in the household	48 (8.3)	41 (7.1)	56 (9.7)	35 (9.2)	25 (6.6)	38 (10.0)
Anthropometric characteristics						
MUAC (mm)	116.0 (112.0, 120.0)	116.0 (111.2, 120.0)	116.0 (112.0, 120.0)	110.0 (105.0, 112.0)	110.0 (105.0, 113.0)	110.0 (106.0, 113.0)
<115 or edema	216 (37.4)	215 (37.5)	215 (37.1)	382 (100.0)	379 (100.0)	379 (100.0)
Nutritional edema						
+	9 (1.6)	8 (1.4)	6 (1.0)	11 (2.9)	9 (2.4)	8 (2.1)
++	3 (0.5)	2 (0.3)	2 (0.3)	3 (0.8)	3 (0.8)	2 (0.5)
Weight (kg)	6.5 (5.8, 7.1)	6.4 (5.7, 7.1)	6.4 (5.8, 7.2)	5.9 (5.2, 6.6)	5.8 (5.2, 6.7)	5.8 (5.3, 6.7)
Height (cm)	70.0 (66.0, 73.5)	69.5 (65.0, 73.5)	69.5 (65.5, 74.0)	68.0 (63.5, 72.0)	67.0 (63.5, 72.0)	67.0 (63.5, 72.2)
WHZ	–2.8 (–3.4, –2.4)	–2.8 (–3.3, –2.3)	–2.7 (–3.3, –2.2)	–3.3 (–3.9, –2.7)	–3.1 (–3.7, –2.7)	–3.1 (–3.7, –2.5)
WHZ category ≥ –2	94 (16.3)	93 (16.2)	127 (21.9)	30 (7.9)	35 (9.2)	50 (13.2)
<–3	220 (38.1)	206 (35.9)	190 (32.8)	222 (58.1)	193 (50.9)	188 (49.6)
HAZ	–3.3 (–4.4, –2.2)	–3.3 (–4.2, –2.3)	–3.3 (–4.2, –2.4)	–3.7 (–4.7, –2.7)	–3.7 (–4.7, –2.8)	–3.6 (–4.6, –2.7)
HAZ category ≥–2	118 (20.4)	101 (17.6)	101 (17.4)	8 (2.1)	3 (0.8)	7 (1.8)
<–3	321 (55.5)	334 (58.2)	329 (56.7)	259 (67.8)	270 (71.2)	266 (70.2)
WAZ	–3.8 (–4.5, –3.1)	–3·8 (–4.4, –3.2)	–3.7 (–4.3, –3.1)	–4.3 (–5.0, –3.7)	–4.3 (–4.9, –3.8)	–4.2 (–4.8, –3.6)
WAZ category ≥–2	18 (3.1)	15 (2.6)	16 (2.8)	24 (6.3)	28 (7.4)	22 (5.8)
<–3	453 (78.4)	454 (79.1)	464 (80.0)	350 (91.6)	348 (91.8)	350 (92.3)
Medical characteristics						
Temperature axillary ≥37.5	78 (13.5)	87 (15.2)	73 (12.6)	56 (14.7)	62 (16.4)	66 (17.4)
Cardiac frequency ≥130 (bpm)	196 (33.9)	164 (28.6)	188 (32.4)	121 (31.7)	106 (28.0)	112 (29.6)
Respiratory frequency ≥40 (bpm)	92 (15.9)	87 (15.2)	70 (12.1)	34 (8.9)	45 (11.9)	28 (7.4)
Oxygen saturation <92 (%)	1 (0.2)	2 (0.3)	2 (0.3)	0 (0.0)	0 (0.0)	1 (0.3)
Positive malaria rapid antigen test performed^1^	135 (23.4)	127 (22.2)	133 (22.9)	139 (36.4)	127 (33.6)	131 (34.6)
Amoxicillin received among children with SAM^2^	297 (100.0)	296 (100.0)	292 (100.0)	382 (100.0)	379 (100.0)	379 (100.0)
Health problem at inclusion	378 (65.4)	394 (68.6)	412 (71.0)	275 (72.0)	265 (69.9)	267 (70.4)
Diarrhea	124 (21.5)	153 (26.7)	158 (27.2)	81 (21.2)	89 (23.5)	87 (23.0)
Respiratory infection	118 (20.4)	96 (16.7)	115 (19.8)	86 (22.5)	68 (17.9)	67 (17.7)
Nutritional supplementation						
Eligible for supplementation^3^	536 (92.7)	572 (99.7)	580 (100.0)	382 (100.0)	379 (100.0)	379 (100.0)
Not eligible for supplementation	42 (7.3)	0 (0.0)	0 (0.0)			
RUSF	238 (41.2)	0 (0.0)	1 (0.2)	1 (0.3)	1 (0.3)	0 (0.0)
RUTF	298 (51.6)	572 (100.0)	579 (99.8)	381 (99.7)	378 (99.7)	379 (100.0)

Abbreviations: Bpm, beats per minute; ComPAS, Combined Protocol for Acute Malnutrition Study; HAZ, height-for-age z**-**score; MUAC, mid-upper arm circumference; OptiMA, Optimizing treatment for acute MAlnutrition; OTP**,** outpatient therapeutic program; RUSF, ready-to-use supplementary food; RUTF, ready-to-use therapeutic food; SAM**,** severe acute malnutrition**;** WAZ, weight-for-age z-score; WHZ, weight for-height z**-score**.

Data are *n* (%) or median (IQR).

1 Two missing data for children with MUAC < 125 mm and 1 missing data for children with MUAC < 115 mm.

2 One missing data for children with MUAC < 125 mm.

3 Two missing data for children with MUAC < 125 mm.

During the study period, 53 children died, including 23 (4.0%), 19 (3.3%, compared with standard arm *P* = 0.54) and 11 (1.9%, compared with standard arm *P* = 0.36) in the standard, OptiMA, and ComPAS arms, respectively (Figure 1). Characteristics of deceased children are presented in Supplemental Table 7. A total of 514 (88.9%), 511 (89.0%), 527 (90.9%) children completed the 6-mo study in the 3 arms (Table 2). The remaining children were either lost to follow-up (31, 35, and 33 in standard, OptiMA, and ComPAS, respectively) or whose families moved out of the study area (10, 9, 9 in standard, OptiMA, and ComPAS, respectively) (Figure 1). Comorbidities were very common and evenly distributed in the 3 groups with 1183 (68.3%), 934 (53.9%), and 989 (57.1%) of children with acute malnutrition having ≥1 episode of malaria, respiratory tract infections, and/or diarrhea during the study period (Table 2). Of the 924 (53.3%) children who met the criteria for hospital referral, only 420 (24.2%) were actually hospitalized with no difference between arms (Table 2).

**TABLE 2:** Follow-up characteristics during the 6-mo trial period (intention-to-treat analysis)

Variable	Children with MUAC < 125 mm or edema (+, ++)					Children with MUAC < 115 mm or edema (+, ++)				*P* value
	Standard, *N* = 578	OptiMA, *N* = 574	*P* value	ComPAS (*N* = 580)	*P* value	Standard (*N* = 382)	OptiMA (*N* = 379)	*P* value	ComPAS (*N* = 379)	
Completed 6-mo follow-up	514 (88.9)	511 (89.0)	0.88	527 (90.9)	0.21	338 (88.5)	312 (82.3)	0.05	326 (86.0)	0.07
Program attendance										
Follow-up visits at the trial center, visits	9.0 (6.0, 15.0)	9.0 (6.0, 15.0)	0.15	9.0 (6.0, 14.0)	0.95	11.0 (8.0, 16.0)	11.0 (7.0, 15.0)	0.44	10.0 (7.0, 15.0)	0.037
Follow-up visits at home, visits	3.0 (2.0, 5.0)	3.0 (2.0, 5.0)	0.40	4.0 (2.0, 5.0)	0.36	3.0 (2.0, 4.0)	3.0 (1.0, 4.0)	0.16	3.0 (2.0, 4.0)	0.70
At least 1 missed visit at health center	239 (41.3)	248 (43.2)	0.52	273 (47.1)	0.050	169 (44.2)	231 (60.9)	<0.001	236 (62.3)	< 0.001
At least 1 missed visit at home	209 (36.2)	214 (37.3)	0.69	213 (36.7)	0.84	131 (34.3)	161 (42.5)	0.020	145 (38.3)	0.26
Comorbidities										
At least 1 health problem during the 6-mo follow-up	561 (97.1)	565 (98.4)	0.12	573 (98.8)	0.038	366 (95.8)	372 (98.2)	0.059	367 (96.8)	0.45
At least 1 RDT malaria positive during the 6-mo follow-up	386 (66.8)	403 (70.2)	0.21	394 (67.9)	0.68	253 (66.2)	266 (70.2)	0.24	255 (67.3)	0.76
At least 1 IRA episode during the 6-mo follow-up	325 (56.2)	312 (54.4)	0.52	297 (51.2)	0.087	230 (60.2)	222 (58.6)	0.65	203 (53.6)	0.064
At least 1 diarrhea episode during the 6-mo follow-up	314 (54.3)	334 (58.2)	0.19	341 (58.8)	0.13	214 (56.0)	218 (57.5)	0.68	222 (58.6)	0.48
At least 1 anemia episode during the 6-mo follow-up	16 (2.8)	14 (2.4)	0.73	26 (4.5)	0.12	9 (2.4)	12 (3.2)	0.50	16 (4.2)	0.15
Hospitalization										
Children with ≥1 follow-up visit with indication for reference to hospital	290 (50.2)	316 (55.1)	0.10	318 (54.8)	0.11	239 (62.6)	247 (65.2)	0.45	234 (61.7)	0.81
Main reason for reference to hospital: stagnant or weight loss	255 (44.1)	284 (49.5)	0.068	277 (47.8)	0.21	212 (55.5)	228 (60.2)	0.19	215 (56.7)	0.73
Children hospitalized at least once	138 (23.9)	142 (24.7)	0.73	140 (24.1)	0.92	92 (24.1)	93 (24.5)	0.88	77 (20.3)	0.21
Main diagnosis – Malaria	75 (54.3)	84 (59.2)	0.42	88 (62.9)	0.15	47 (51.1)	52 (55.9)	0.51	50 (64.9)	0.070
MUAC at hospitalization (mm)	115.0 (110.0, 120.0)	115.0 (110.0, 120.0)	0.32	115.0 (110.8, 120.0)	0.64	110.0 (105.0, 115.2)	111.0 (105.0, 116.0)	0.76	112.0 (107.0, 116.0)	0.27
Nutritional edema at hospitalization	4 (2.9)	5 (3.5)	>0.99	7 (5.0)	0.37	1 (1.1)	1 (1.1)	>0.99	5 (6.5)	0.094
Length of therapeutic milk F-75 received (d)	2.0 (2.0, 3.0)	2.0 (2.0, 3.0)	0.47	2.0 (1.0, 3.0)	0.20	2.0 (1.0, 2.0)	2.0 (2.0, 3.0)	0.14	2.0 (1.0, 3.0)	0.40
Length of therapeutic milk F-100 received (d)	1.0 (0.0, 1.0)	1.0 (1.0, 2.0)	0.041	1.0 (1.0, 2.0)	0.025	1.0 (0.0, 1.0)	1.5 (1.0, 2.0)	0.053	1.0 (1.0, 2.0)	0.16
Length of RUTF received (d)	2.0 (2.0, 3.0)	2.0 (2.0, 3.0)	0.059	2.0 (2.0, 3.0)	0.57	2.0 (2.0, 3.0)	2.0 (2.0, 3.0)	0.73	2.0 (2.0, 3.0)	0.61
MUAC at the end of hospitalization^1^ (mm)	117.0 (112.0, 123.0)	116.0 (112.0, 120.0)	0.32	117.0 (114.0, 122.0)	0.87	112.0 (105.0, 117.2)	113.0 (108.0, 117.0)	0.50	114.0 (107.5, 120.0)	0.24
Length of hospitalization^1^ (d)	4.0 (3.0, 5.0)	4.0 (3.0, 6.0)	0.95	4.0 (3.0, 5.0)	0.95	4.0 (3.0, 6.0)	5.0 (3.0, 6.0)	0.49	5.0 (3.0, 6.0)	0.30

Data are median (IQR) or *n* (%).

Abbreviations: ARI, Acute Respiratory Infection; ComPAS, Combined Protocol for Acute Malnutrition Study; HAZ, height-for-age z-score; MUAC, mid-upper arm circumference; OptiMA, Optimizing treatment for acute MAlnutrition; RDT, Rapid Diagnostic Test; RUSF, ready-to-use supplementary food; RUTF, ready-to-use therapeutic food; WAZ, weight-for-age z-score; WHZ, weight for-height z-score.

1 Calculated at first hospitalization (128 in the standard group, 139 in the OptiMA group, 136 in the ComPAS group).

**FIGURE 2:**
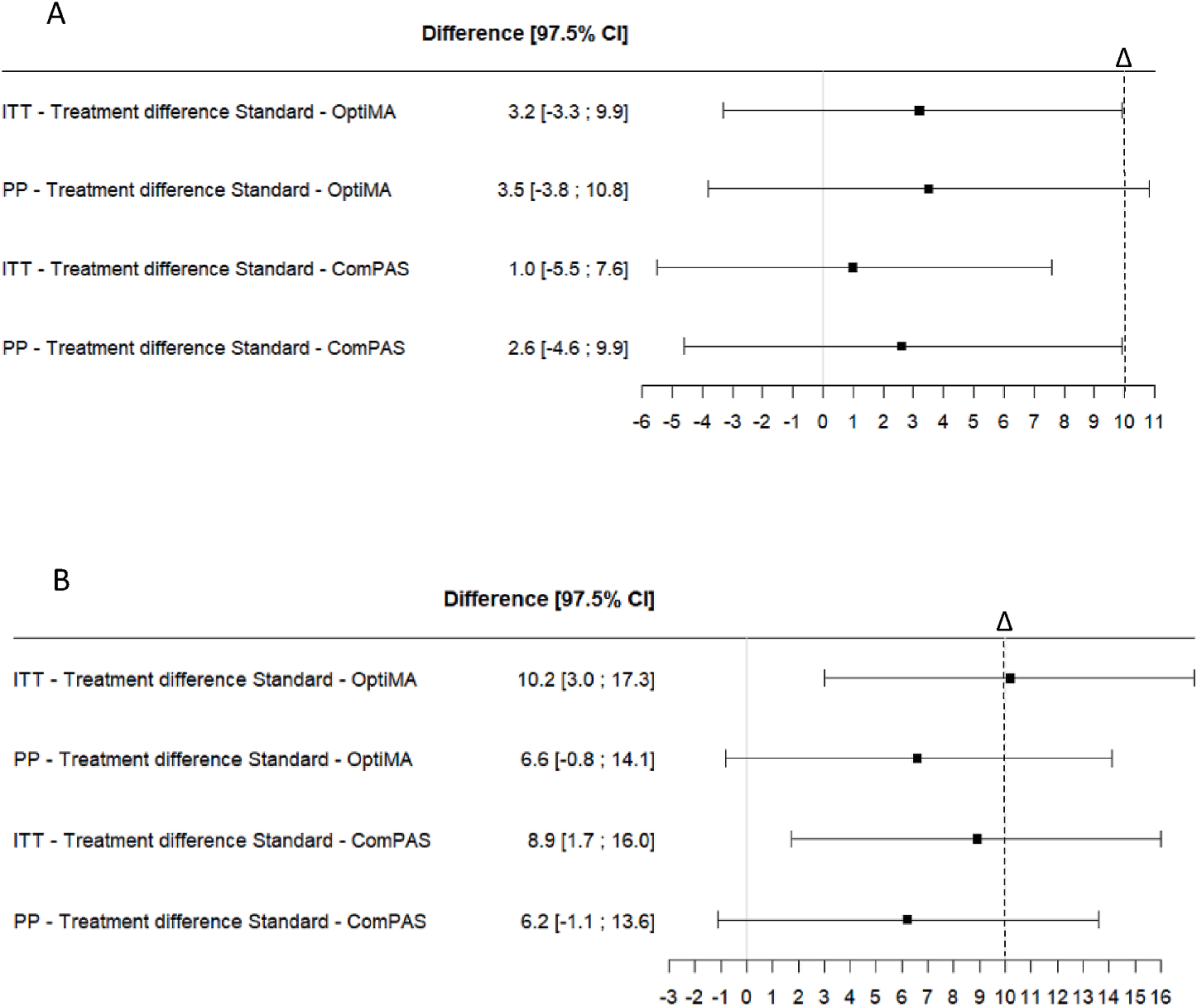
Intention-to-treat (ITT) and per-protocol (PP) noninferiority analysis of the primary outcome and nutritional recovery for the main secondary objective. (A) Primary outcome among children admitted with mid-upper arm circumference (MUAC) < 125 mm or edema (*N* = 1732), assessed 6 mo after inclusion: child alive, not acutely malnourished (MUAC ≥125 mm, no bilateral nutritional edema, and weight-for-height z-score ≥ −3), and not having experienced any new episode of acute malnutrition over the 6-mo observation period. (B) Nutritional recovery outcome among children with MUAC < 115 mm assessed over the 6-mo follow-up trial period: after a 4-wk minimum duration of supplementation, an axillary temperature <37.5◦C, MUAC ≥125 mm and no bilateral nutritional edema for 2 consecutive weeks. PP definition: minimum prescription of 4 wk of supplementation, children received ≥90% of the total amount of supplementation they were supposed to receive as per protocol and a maximum interval of 8 wk between any 2 visits in the 6-mo follow-up. Δ, noninferiority margin (=10%).

The proportion of children achieving the primary outcome was lower than expected (68%) with no significant difference between the 3 arms, 3% and 1% of difference were found between OptiMA and ComPAS compared with the standard arm, respectively (Table 3). The main cause of not achieving the primary outcome was relapse to another episode of acute malnutrition with no difference between the 3 arms (Table 3). Noninferiority was shown in ITT analysis for both OptiMA and ComPAS and in PP analysis for ComPAS only (Figure 2).

**TABLE 3:** Primary outcome at 6 mo and nutritional recovery over 6-mo follow-up

Variable	Children with MUAC < 125 mm or edema (+,++)				Children with MUAC < 115 mm or edema (+, ++)		
	Standard	OptiMA	ComPAS		Standard	OptiMA	ComPAS
Intention-to-treat analysis							
Number of patients	578	574	580	Number of patients	382	379	379
Achieving primary outcome^1^	306 (52.9)	285 (49.7)	301 (51.9)	Nutritional recovery^2^	299 (78.3)	258 (68.1)	263 (69.4)
Not achieving primary outcome				No nutritional recovery			
Death	23 (4.0)	19 (3.3)	11 (1.9)	Death^3^	12 (3.1)	8 (2.1)	5 (1.3)
Discontinued trial^4^	41 (7.1)	44 (7.7)	42 (7.2)	Discontinued trial^4^	11 (2.9)	22 (5.8)	23 (6.1)
Initial acute malnutrition episode unresolved at 6 mo	39 (6.7)	46 (8.0)	43 (7.4)	MUAC ≥ 125 mm and no edema 1 wk only	12 (3.1)	31 (8.2)	29 (7.7)
New episode of acute malnutrition resolved at 6 mo	141 (24.4)	133 (23.2)	141 (24.3)	Never reached MUAC ≥ 125 mm and no edema	45 (11.8)	50 (13.2)	46 (12.1)
New episode of acute malnutrition unresolved at 6 mo	28 (4.8)	47 (8.2)	42 (7.2)	RUTF <4 visits	3 (0.8)	10 (2.6)	13 (3.4)
Per-protocol analysis^5^							
Number of patients	451	484	489	Number of patients	323	293	298
Achieving primary outcome^1^	260 (57.6)	262 (54.1)	269 (55.0)	Nutritional recovery 2	265 (82.0)	221 (75.4)	226 (75.8)
Not achieving primary outcome				No nutritional recovery			
Death	23 (5.1)	19 (3.9)	11 (2.2)	Death	12 (3.7)	8 (2.7)	5 (1.7)
Initial acute malnutrition episode unresolved at 6 mo	30 (6.7)	40 (8.3)	40 (8.2)	MUAC ≥ 125 mm and no edema 1 wk only	10 (3.1)	22 (7.5)	19 (6.4)
New episode of acute malnutrition resolved at 6 mo	117 (25.9)	121 (25.0)	130 (26.6)	Never reached MUAC ≥ 125 mm and no edema	36 (11.1)	37 (12.6)	39 (13.1)
New episode of acute malnutrition unresolved at 6 mo	21 (4.7)	42 (8.7)	39 (8.0)	RUTF <4 visits	0 (0.0)	5 (1.7)	9 (3.0)

Data are *n* (%), unless stated otherwise.

Abbreviations: ComPAS, Combined Protocol for Acute Malnutrition Study; MUAC, mid-upper arm circumference; OptiMA, Optimizing treatment for acute MAlnutrition; RUTF, ready-to-use therapeutic food.

1 Assessed 6-mo after inclusion: child alive, not acutely malnourished (MUAC ≥ 125 mm, no bilateral nutritional edema, and weight-for-height z-score ≥ −3), and not having experienced any new episode of acute malnutrition over the 6-mo observation period.

2 Assessed over the 6-mo follow-up trial period: after a 4-wk minimum duration of supplementation, an axillary temperature <37.5◦C, MUAC ≥ 125 mm and no bilateral nutritional edema for 2 consecutive weeks.

3 Eight children met the nutritional recovery criteria before death.

4 Family moved out of the study area or children were lost to follow-up at 6 mo.

5 Per-protocol definition: minimum prescription of 4 wk of supplementation, children received ≥90% of the total amount of supplementation they were supposed to receive as per protocol and a maximum interval of 8 wk between any 2 visits in the 6-mo follow-up.

There was no difference between the 3 arms among acute malnutrition children in terms of weight, MUAC, and height gains at 6 mo post randomization (Table 4). There was also no difference found in nutritional recovery between the standard (83.4%) and ComPAS arms (80.2%, *P* = 0.16) whereas fewer children recovered in the OptiMA group (77.9%, *P* = 0.018). During the 6-mo follow-up, children in the standard arm received a median of 111 sachets (IQR: 49–200) of RUTF and/or RUSF over a 9-wk period (IQR: 6–14) (Table 4). In comparison, those in the OptiMA arm received a median of 94 sachets (IQR: 53–158) over 10 wk (IQR: 6–15), and children in the ComPAS arm received 70 sachets (IQR: 42–105) over 9 wk (IQR: 6–14) (Table 4).

**TABLE 4:** Nutritional and anthropometric characteristics over 6 mo post inclusion (intention-to-treat analysis)

Variable	Children with MUAC <125 mm or edema (+, ++)					Children with MUAC <115 mm or edema (+, ++)				
	Standard (*N* = 578)	OptiMA (*N* = 574)	*P* value	ComPAS (*N* = 580)	*P* value	Standard (*N* = 382)	OptiMA (*N* = 379)	*P* value	ComPAS (*N* = 379)	*P* value
Anthropometric characteristics at 6 mo										
MUAC (mm)	130·0 (125.0, 135.0)	130.0 (125.0, 135.0)	0.29	130.0 (125.0, 135.0)	0.62	130.0 (125.0, 135.0)	127.0 (122.0, 133.5)	0.004	129.0 (123.0, 135.0)	0.32
MUAC < 115 mm or edema	25 (4.3)	22 (3.8)	0.20	23 (4.0)	0.60	32 (8.4)	25 (6.6)	0.002	27 (7.1)	0.084
Weight (kg)	8.1 (7.4, 9.0)	8.0 (7.2, 9.0)	0.13	8.2 (7.5, 9.0)	0.80	8.0 (7.2, 8.9)	7.7 (6.9, 8.7)	0.019	7.9 (7.1, 8.8)	0.46
Height (cm)	72.0 (68.5, 76.0)	72.0 (68.0, 76.0)	0.46	72.2 (68.3, 76.5)	0.73	70.4 (67.0, 75.0)	69.5 (66.2, 74.4)	0.25	69.6 (65.8, 75.0)	0.43
WHZ	–0.7 (–1.5, 0.0)	–0.8 (–1.5, –0.1)	0.37	–0.7 (–1.4, 0.0)	0.69	–0.4 (–1.3, 0.4)	–0.6 (–1.5, 0.1)	0.039	–0.5 (–1.2, 0.4)	0.76
WHZ <–3	23 (4.0)	21 (3.7)	0.54	18 (3.1)	0.30	24 (6.3)	15 (4.0)	0.048	18 (4.7)	0.48
HAZ^1^	–4.0 (–4.8, –3.1)	–4.0 (–4.8, –3.2)	0.90	–4.0 (–4.6, –3.3)	0.63	–4.5 (–5.3, –3.6)	–4.5 (–5.2, –3.7)	0.57	–4.4 (–5.1, –3.7)	0.71
HAZ <–3^1^	452 (78.2)	455 (79.3)	0.45	466 (80.3)	0.65	324 (84.8)	336 (88.7)	0.10	338 (89.2)	0.20
WAZ^1^	–2.8 (–3.5, –2.0)	–2.7 (–3.5, –2.0)	0.54	–2·7 (–3.3, –2.0)	0.45	–2.8 (–3.6, –2.0)	–3.0 (–3.7, –2.2)	0.044	–2.8 (–3.5, –2.1)	0.88
WAZ <–3^1^	231 (40.0)	238 (41.5)	0.87	218 (37.6)	0.43	163 (42.7)	190 (50.1)	0.021	164 (43.3)	0.057
Change in anthropometric parameters between inclusion and 6 mo										
Duration in the trial (wk)	26.0 (25.6, 26.3)	26.0 (25.6, 26.3)	0.36	26.0 (25.7, 26.3)	0.42	26.0 (25.6, 26.1)	25.9 (25.3, 26.1)	0.064	26 (25, 26)	0.98
MUAC gain (mm)	14 (8, 20)	14 (9, 20)	0.80	14 (9, 20)	0.66	21 (14, 27)	19 (13, 25)	0.005	20 (13, 26)	0.25
Weight gain (g)	1800 (1200, 2400)	1700 (1200, 2200)	0.19	1800 (1300, 2300)	0.97	2100 (1500, 2700)	1900 (1250, 2500)	<0.001	2000 (1450, 2600)	0.22
Height gain (cm)	2.5 (1.5, 3.5)	2.5 (1.6, 3.5)	0.22	2.5 (1.7, 3.5)	0.067	2.3 (1.5, 3.2)	2.2 (1.5, 3.2)	0.80	2.5 (1.5, 3.5)	0.48
Daily weight gain (g/kg/d)	1.6 (1.1, 2.2)	1.5 (1.1, 2.1)	0.44	1.6 (1.1, 2.0)	0.39	2.1 (1.5, 2.8)	2.0 (1.3, 2.7)	0.043	2.0 (1.5, 2.7)	0.10
Weekly MUAC gain (mm/wk)	0.6 (0.4, 0.8)	0.6 (0.4, 0.8)	0.94	0.6 (0.4, 0.8)	0.78	0.9 (0.6, 1.1)	0.8 (0.6, 1.1)	0.10	0.8 (0.6, 1.1)	0.18
Nutritional improvement										
Children who recovered during the 6-mo follow-up^2^	482 (83.4)	447 (77.9)	0.018	465 (80.2)	0.16	299 (78.3)	258 (68.1)	0.001	263 (69.4)	0.005
Time to reach recovery^2^ by 6 mo (wk)	8.0 (4.0, 12.0)	7.0 (4.0, 12.0)	0.38	7.0 (4.0, 13.0)	0.65	10.0 (7.0, 14.5)	11.0 (7.0, 16.0)	0.20	11.0 (7.0, 17.0)	0.12
Children who recovered^2^ by 12 wk	363 (62.8)	341 (59.4)	0.24	348 (60.0)	0.33	193 (50.5)	149 (39.3)	0.002	155 (40.9)	0.008
Children who recovered^2^ by 16 wk	414 (71.6)	376 (65.5)	0.025	393 (67.8)	0.15	249 (65.2)	200 (52.8)	<0.001	194 (51.2)	<0.001
Nutritional recovery^3^ 1 week only	511 (88.4)	506 (88.2)	0.89	517 (89.1)	0.69	314 (82.2)^4^	300 (79.2)^4^	0.29	304 (80.2)^4^	0.48
Achieving primary outcome^5^	—	—		—		228 (59.7)	200 (52.8)	0.055	213 (56.2)	0.33
Nutritional treatment distributed										
Children not eligible for supplementation	42 (7.3)	0 (0.0)	<0.001	0 (0.0)	<0.001	0 (0.0)	0 (0.0)		0 (0.0)	
Median amount of RUTF/RUSF received over the 6-mo follow-up, sachet	111.0 (49.0, 200.0)	94.0 (53.0, 158.0)	0.014	70.0 (42.0, 105.0)	<0.001	185.0 (130.0, 247.0)	126.0 (80.0, 183.0)	<0.001	91.0 (63.0, 140.0)	<0.001
Median length of RUTF/RUSF treatment over the 6-mo follow-up (d)	63.0 (42.0, 98.0)	70.0 (42.0, 105.0)	0.13	63.0 (42.0, 98.0)	0.92	77.0 (56.0, 112.0)	77.0 (49.0, 106.0)	0.63	70.0 (49.0, 105.0)	0.082
Median amount of RUTF/RUSF received during the initial episode, sachet	98.0 (40.5, 175.0)	73.0 (44.0, 140.0)	0.058	56.0 (35.0, 91.0)	<0.001	170.0 (120.0, 233.8)	121.0 (77.0, 180.5)	<0.001	84.0 (56.0, 133.0)	<0.001
Median length of RUTF/RUSF treatment during the initial episode (d)	56.0 (35.0, 84.0)	56.0 (35.0, 91.0)	0.14	49.0 (35.0, 77.0)	0.74	70.0 (49.0, 98.0)	77.0 (49.0, 105.0)	0.74	70.0 (49.0, 101.5)	0.13
Energy received during the initial episode (kcal/kg/d)	170.2 (86.3, 188.0)	106.9 (87.2, 131.3)	<0.001	83.8 (73.8, 103.0)	<0.001	181.9 (176.3, 190.5)	131.5 (116.2, 153.8)	<0.001	105.8 (90.5, 133.8)	<0.001

Data are median (IQR) or *n* (%).

Abbreviations: ComPAS, Combined Protocol for Acute Malnutrition Study; HAZ, height-for-age z-score; MUAC, mid-upper-arm circumference; OptiMA, Optimizing treatment for acute MAlnutrition; RUSF, ready-to-use supplementary food; RUTF, ready-to-use therapeutic food; WAZ, weight-for-age z-score; WHZ, weight for-height z-score.

1 One child aged >59 mo was excluded from this analysis.

2 Assessed after a 4-wk minimum duration of supplementation, an axillary temperature <37.5◦C, MUAC ≥125 mm and no bilateral nutritional edema for 2 consecutive weeks.

3 Assessed over the 6-mo follow-up trial period defined as reaching MUAC ≥125 mm and no bilateral nutritional edema.

4 Three children who reached a MUAC ≥125 mm without edema were classified as deceased and 23 as “RUTF <4 visits” in Table 3.

5 Assessed 6-mo after inclusion: child alive, not acutely malnourished (MUAC ≥ 125 mm, no bilateral nutritional edema, and weight-for-height z-score ≥ −3), and not having experienced any new episode of acute malnutrition until at least a 6-mo post-enrollment follow-up.

**FIGURE 3:**
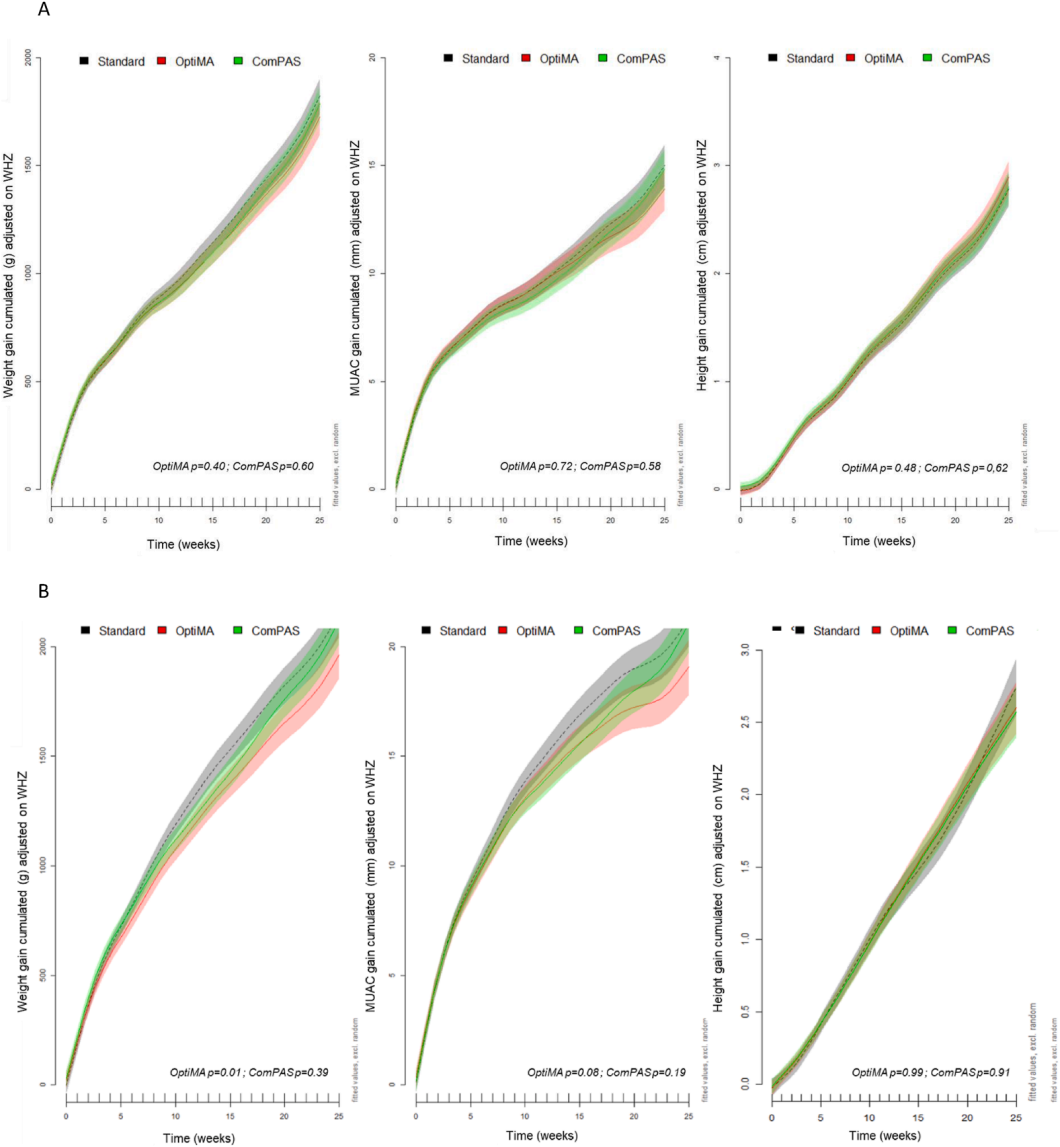
Modeled adjusted weekly means of weight, MUAC and height cumulated gains over 6 mo by randomization groups (intention-to-treat). (A) Children admitted with MUAC < 125 mm or edema. (B) Children admitted with MUAC < 115 mm or edema. ComPAS, Combined Protocol for Acute Malnutrition Study; MUAC, mid-upper-arm circumference; OptiMA, Optimizing treatment for acute MAlnutrition; WHZ, weight for-height z-score.

In post-hoc analyses, growth curves adjusted for age, sex, and WHZ at admission were similar in the 3 arms for MUAC, weight, and height gains, with the 95% CI overlapping throughout the 6-mo period. For better readability, Figure 3 only shows growth curves adjusted on WHZ as this variable was not similarly distributed in the 3 arms. Growth curves adjusted on age and sex are presented in Supplemental Figure 2.

### Results for children randomly assigned with MUAC < 115 mm or edema

Baseline sociodemographic, anthropometric and clinical characteristics were similar in the 3 arms (Table 1) except for children with WHZ ≥–2 at admission, which was 30 (7.9%), 35 (9.2%), 50 (13.2%) in the standard, OptiMA and ComPAS arms, respectively. The median age was 14 mo (IQR: 9–22), 644 (56.5%) of all the children were girls, and the median MUAC at admission was 110 mm (IQR: 105–112) for the 3 arms.

The 6-mo follow-up was completed for 338 (88.5%), 312 (82.3%), and 326 (86.0%) children, 14 (3.7%), 13 (3.4%), and 6 (1.6%) children died and 25 (6.5%), 45 (11.9%) and 41 (10.8%) children were lost to follow-up at 6-mo post randomization in the standard, OptiMA and ComPAS arms, respectively (Figure 1; Table 2). Children were more likely to miss clinic visits in the intervention arms, with 231 (60.9%) in OptiMA, 236 (62.3%) in ComPAS, and 169 (44.2%) in the standard arm (Table 2).

For children included with MUAC < 115 mm or edema, the standard protocol was superior in ITT analysis and noninferiority was not demonstrated in PP for both OptiMA and ComPAS. (Figure 2). This difference was largely driven by the higher number of children who recorded only 1 wk of MUAC > 125 mm and no edema in the intervention arms, with 31 children (8.2%) in OptiMA, 29 (7.7%) in ComPAS and 12 (3.1%) in the standard arm (Table 3). When looking at children who reach a MUAC > 125 mm without edema for 1 wk, there was no significant difference across arms, with 314 (82.2%) children in the standard, 300 (79.2%) in OptiMA, and 304 (80.2%) children in ComPAS (Table 3). A noninferiority sensitivity analysis for these children is presented in Supplemental Tables 8 and 9 and Supplemental Figure 3. This analysis showed that both OptiMA and ComPAS arms are noninferior to the standard protocol when 1-wk recovery was the defined endpoint.

At 6 mo post randomization, there was no evidence of a difference between the standard and ComPAS arms in terms of median MUAC gain (21 mm, IQR: 14–27 compared with 20 mm, IQR: 13–26, *P* = 0.25) and weight gain (2.1 kg, IQR: 1.5–2.7 compared with 2.0 kg IQR: 1.4–2.6, *P* = 0.22) whereas the OptiMA group had lower anthropometric parameters (Table 4). During the initial episode, children in the standard arm received a median of 170 sachets of RUTF (IQR: 120.0–233.8) over a 10-wk period (IQR: 7–14) (Table 4). In comparison, those in the OptiMA arm received a median of 121 sachets (IQR: 77.0–180.5) over 11 wk (IQR: 7–15), and children in the ComPAS arm received 84 sachets (IQR: 56.0–133.0) over 10 wk (IQR: 7–14.5) (Table 4).

In post-hoc analysis, growth curves adjusted for age, sex, and WHZ at admission showed similar cumulative weight, MUAC, and height gains in the standard and ComPAS arms throughout the 6-mo period (Supplemental Figures 2 and 3). During the first weeks of treatment, the 95% CI of the cumulative weight gain overlapped for all 3 arms, but from week 8 to exit, children randomly assigned to the OptiMA arm gained weight more slowly (Figure 3).

## Discussion

This trial evaluated the noninferiority of 2 simplified strategies, OptiMA and ComPAS, compared with the national standard protocol for the management of acute malnutrition in children aged 6–59 mo in Niger. Among children with MUAC < 125 mm or edema, noninferiority in terms of being alive and achieving and maintaining a MUAC ≥ 125 mm, WHZ ≥−3, and no edema over 6 mo was demonstrated in ComPAS for both ITT and PP analyses and only in ITT analysis for OptiMA. Secondary outcomes, including mortality, hospitalization, anthropometric indicators, and 6-mo growth trajectories, were comparable across all arms, despite a 37% reduction in RUTF use in ComPAS and a 15% reduction in OptiMA. Among children included with MUAC < 115 mm or edema, the standard protocol was superior in ITT analysis in terms of nutritional recovery. Mortality and hospitalizations were similar across arms, whereas nutritional status and growth trajectories over 6 mo were comparable between standard and ComPAS but lower for OptiMA. Among these children, ComPAS and OptiMA used 50% and 32% less RUTF, respectively, compared with the standard. Importantly, a post-hoc analysis showed noninferiority of both intervention arms among children who achieved 1 wk of recovery instead of 2 and nearly 20% more missed visits in the intervention arms.

The proportion of children achieving the primary outcome measure was lower than anticipated in all arms (we expected 68%), and led to a lack of statistical power for demonstrating noninferiority for the OptiMA arm in PP analysis. This was likely due to more severe malnutrition at inclusion than expected and the high proportion of comorbidities during follow-up, probably exacerbated by measles and COVID-19 outbreaks, as well as a national RUTF shortage before the start of enrollments. Notably, there was no significant difference in the absolute percentage of children achieving the primary outcome across the 3 arms.

For the main secondary outcome, the standard protocol demonstrated superiority in the ITT analysis in terms of nutritional recovery at 2 consecutive visits for children with MUAC < 115 mm or edema at admission. However, a post-hoc analysis showed no difference across the 3 arms for children meeting recovery criteria for 1 wk only, suggesting that it was the more stringent 2-visit criterion that was responsible for both intervention arms not being noninferior to the standard. As the standard protocol has been implemented for 20 y in the Zinder region, achieving adherence to the simplified protocol with a reduced RUTF dosage was a major challenge. Missed clinic visits were more frequent in the simplified protocol arms, despite discharge rations and soap given to mothers as incentives, suggesting that the additional RUTF sachets in the standard arm may be used by families to reduce opportunity costs (like transport to the health center). Exploring more efficient ways to reduce these costs is crucial to increase the effectiveness and coverage of these programs [24,25]. Recent studies explored operational solutions to scale up simplified approaches, such as the screening and treatment of acute malnutrition by CHWs [[26], [27], [28]] or reducing the frequency of outpatient visits [29,30].

The absence of significant differences in the ComPAS arm in secondary outcomes, including mortality and hospitalization proportions, absolute MUAC, weight and height gain at 6 mo, anthropometric growth over 6 mo suggests that reduced RUTF dosages do not negatively impact long-term health outcomes. This finding aligns with previous randomized clinical trials (RCTs) in other settings, which have shown that reduced dosages do not increase the risk to children [11,13,15]. It is perplexing that the OptiMA arm, among children admitted with MUAC < 115 mm or edema, exhibited significant differences in anthropometric outcomes than the standard arm. We cannot entirely exclude that it was not related to the individual randomization or to the open status design. The simplicity of the ComPAS strategy (2 sachets/d for MUAC < 115 mm or edema and 1 sachet/d for children with MUAC 115–124 mm) may also have made it easier for families to follow, potentially improving compliance. These findings contrast with those of the OptiMA trial conducted in the DRC, where the OptiMA protocol, using the same methodology, demonstrated superiority among children with MUAC < 125 mm or edema, and noninferiority among children with SAM (as defined by WHO) compared with the national protocol [15,16]. For over 20 y, populations in Niger have been accustomed to receiving the quantities specified in the standard protocol whereas in the OptiMA-DRC study area the standard protocol was only introduced for the first time in 2018. These differences highlight how the implementation and adherence to simplified protocol can be highly context-dependent.

This is the first study to evaluate 2 reduced-dose RUTF regimens in comparison to a standard protocol in an individually randomized study design. Our findings suggest that similar growth is possible with smaller rations of therapeutic foods with no significant impact from the degree of ration tapering. The weight and MUAC growth over 6 mo, as well as the absolute weight and MUAC gain at 6 mo, were similar between the standard and ComPAS arms in both populations, despite significantly lower RUTF dosages in ComPAS. In contrast, children randomly assigned in the OptiMA arm showed lower anthropometric outcomes, despite receiving higher nutritional intake compared with those in the ComPAS arm. Among children with MUAC < 115 or edema at admission, the standard arm received a median of 170.0 kcal/kg/d (IQR: 120.0–233.8) during the initial episode with a median length of treatment of 10 wk (IQR: 7–14), whereas the ComPAS group received 172.4 kcal/kg/d (IQR: 151.5–190.5) for 2 wk and then 7 wk at 76.6 kcal/kg/d (IQR: 68.5–83.8). In line with a recent viewpoint [31], these findings challenge the high caloric rations currently specified in the last WHO recommendations which suggest an energy requirement of 150–185 kcal/kg/d until a child is no longer SAM, after which the ration can be reduced to 100–130 kcal/kg/d until full anthropometric recovery [8].

One cannot exclude more intensive care in the context of this RCT, which could have led to high hospital admission and the availability of thorough medical evaluation for nonresponsive participants. This might have influenced study outcomes. However, this was a trial conducted in real programmatic settings. Additional staffing at health centers was limited to 1 clinical trial technician per site, with regular trained Ministry of Health (MoH) nurses and routine monitoring staff involved. The high hospitalization proportion is likely more reflective of the more severe nutritional status of enrolled children, rather than an intensification of care procedures. Importantly, hospitalization proportions were similar across all study arms, suggesting that this aspect of care did not differentially affect the outcomes between groups. Although it is possible that access to medical evaluation may have improved outcomes, we believe this potential effect would have been similar across all arms, and thus is unlikely to have biased the demonstration of noninferiority.

Our trial also provides evidence to the updated WHO recommendation suggesting prolonged treatment to ensure both MUAC ≥125 mm and WHZ ≥–2 for recovery. Among children admitted with MUAC < 125 mm, those treated under simplified protocols, using MUAC-only discharge criteria, showed similar anthropometric status at 6 mo, relapse proportions, and durations of supplementation compared with children discharged under the standard protocol applying both MUAC and WHZ criteria. Similar findings were observed among children with MUAC < 115 mm or edema, particularly between the ComPAS and standard arms. These results suggest that requiring both MUAC and WHZ for discharge may not offer additional clinical benefit, whereas MUAC-based protocols potentially offer advantages in terms of workload optimization, resource allocation, and time available for patient-centered care. MUAC > 125 mm has been strongly associated with a lower risk of mortality, further supporting its adequacy as a standalone discharge criterion [[32], [33], [34]]. A dedicated secondary analysis focusing on discharge criteria and relapse is underway and will provide further insights into this important topic.

Finally, this study underscores the balance between individual and collective benefits in managing acute malnutrition. Although the individual clinical advantages of reducing RUTF dosages seem minimal, the collective gains could be substantial. Strategies like OptiMA and ComPAS, by optimizing resource use and simplifying treatment processes, have the potential to increase program coverage, particularly in high-burden settings. This trial strictly adhered to the Nigerien standard protocol, which uses RUTF to treat SAM and RUSF to treat moderate acute malnutrition. However, in real-world conditions where programs are managed by different actors with parallel supply chains, many programs are unable to provide RUSF for moderate acute malnutrition treatment. Understanding the cost of treatment and its drivers is essential for determining the most cost-efficient strategies, but existing evidence is limited, difficult to compare, and highly context-dependent, emphasizing the need for more robust cost data across diverse settings [5,[35], [36], [37]]. An ongoing cost-effectiveness analysis using data from the OptiMA Niger trial will further explore the possible public health benefits of simplified strategies compared with the standard protocol.

The main strength of this study was conducting a clinical trial in a rural region with one of the highest incidences of wasting worldwide and tough security constraints, while maintaining high data quality and ensuring minimal missing data throughout the 6-mo follow-up. The proportions of loss to follow-up and defaulters among all children with acute malnutrition were low (<8%), even though the study population resided in a rural area. Another limitation not previously discussed is the open-label design, which could introduce bias. Mothers familiar with the standard protocol may have been able to know whether their child was randomly assigned to the reduced-dose protocols which could explain the lack of adherence. A cluster design may have mitigated lower compliance with the reduced-dose protocols in a setting where acute malnutrition programs are well known, but we would then have lost the strength of the individual randomization.

Future research should aim to address several key issues: first, the criteria for defining nutritional recovery in acute malnutrition trials should be revisited. The WHO criteria, which require a second recovery visit, were originally introduced to prevent prematurely ending treatment due to measurement errors, without any clinical assumption. A more pragmatic approach, focusing on long-term growth outcomes may be more appropriate for evaluating the real-world effectiveness of simplified protocols. Second, studies are needed on the acceptability of simplified protocols at the community level and among health workers, as well as studies determining the impact of the simplified strategies in terms of treatment coverage and reduction in the burden of malnutrition over time. Lastly, trials should continue to explore the cost-effectiveness of these strategies, given their potential to significantly reduce RUTF consumption while maintaining good health outcomes.

This study provides important insights into the potential of simplified treatment protocols for acute malnutrition and highlights the importance of context in determining program effectiveness. Among children with MUAC < 125 mm or edema, the ComPAS protocol demonstrated noninferiority in both ITT and PP analysis in terms of the primary outcome, whereas OptiMA only demonstrated noninferiority in ITT analysis. OptiMA’s lower performance is unclear, but that there was no difference in growth at 6 mo between standard and ComPAS suggests reduced RUTF dosage had limited influence on overall child growth, as ComPAS used less RUTF than OptiMA. For the most malnourished children with MUAC < 115 mm or edema, the standard protocol showed a higher recovery proportion, but primarily due to better attendance rather than differences in mortality or growth at 6 mo. The potential for cost savings and increased program coverage supports further investigation, focusing on the individual and public health benefits of these simplified strategies to optimize the management of acute malnutrition, especially in resource-limited settings.

## Author contributions

The authors’ responsibilities were as follows – SS, RB: developed the clinical and methodological study concept and had final responsibility for the decision to submit the manuscript for publication; RB, MD, CC, SS: designed the study methodology and wrote the protocol; MD, JH, HB, AAGMA, BM: coordinated the study teams; SA: coordinated the Ministry of Health of Niger staff working in the trial; J-CA, J-JK, BS, JH, HB, MD, AB: organized and supervised data collection; MD, CC, VJ, XA, SS, RB: developed the statistical analysis strategy; MD, VJ: performed the statistical analysis and all coauthors interpreted the results; MD: wrote the first draft of the manuscript with substantial inputs from SS, KP, SK, CC, RB, XA; SS, RB: primarily responsible for the final content of the manuscript; and all authors: had full access to all the data in the study, read and approved the final version.

## Data availability

In accordance with the International Committee of Medical Journal Editors, the data generated by the trial will be made available starting 6 mo and ending 36 mo following the article publication. After this timepoint, the data will be available on request to the corresponding author to researchers who can demonstrate a methodologically sound proposal and whose proposed use of the data has been approved by an independent review committee.

## Funding

The study is funded by the Research for Health in Humanitarian crises at Elrha (www.elrha.org), a global charity that finds solutions to complex humanitarian problems through research and innovation (R2HC, grant number 40344), the Givewell foundation (Oakland, US, www.givewell.org/) and received additional funding from European Commission through the European Civil Protection and Humanitarian Aid Operations (ECHO, https://civil-protection-humanitarian-aid.ec.europa.eu, grant number ECHO/-AF/BUD/2021/92068). This article covers humanitarian aid activities implemented with the financial assistance of the EU. The views expressed herein should not be taken, in any way, to reflect the official opinion of the EU, and the European Commission is not responsible for any use that might be made of the information it contains. The funders had no role in study design, data collection and analysis, decision to publish, or preparation of the manuscript.

## Conflict of interest

KP reports a relationship with Nutriset SAS that includes: consulting or advisory. All other authors report no conflicts of interest.
