# Supplementary material for: Optimizing management of uncomplicated acute malnutrition in children in rural Niger: a 3-arm individually randomized, controlled, noninferiority trial
Source: Am J Clin Nutr. 2025 Sep 1;122(4):972–85. doi: 10.1016/j.ajcnut.2025.07.004 (PMC12674072; doi:10.1016/j.ajcnut.2025.07.004)
Supplement: Supplementary file 1 [file mmc1.docx]

**Supplementary material**

**Title:** Optimizing management of uncomplicated acute malnutrition in children in rural Niger: a 3-arm individually randomized, controlled, non-inferiority trial

**First author:** Maguy Daures

**Supplementary Tables**

**Supplementary Table 1: Wasting definition, treatment products, calculation of dosage and recovery definition in the Niger national, OptiMA and ComPAS protocols**

|  | **National Niger protocol** | | |  | **OptiMA protocol** | | |  | **ComPAS protocol** | |
| --- | --- | --- | --- | --- | --- | --- | --- | --- | --- | --- |
|  | **SAM** | **MAM** | |  | **Acute Malnutrition** | | |  | **Acute Malnutrition** | |
| *Wasting definition* | MUAC<115mm or WHZ<-3 or bipedal edema | MUAC (115–124 mm)  or −3 <WHZ <−2 and  without bipedeal edema | |  | MUAC<125 mm or bipedal edema | | |  | MUAC<125 mm or bipedal edema | |
|  |  |  |  |  | MUAC<115mm or bipedal edema | MUAC 115– 119 mm | MUAC 120– 124 mm |  | MUAC<115mm or bipedal edema | MUAC 115 – 124 mm |
| *Treatment product and quantity* | RUTF 150–200 kcal/ kg/day | If aged 6-23 months : Super cereal plus 200 g/day (~787 kcal/day) or RUSF, one 92 g sachet/day (500kcal/day) | If aged 24-59 months : no supplementation |  | RUTF 170–200 (kcal/kg/day) | RUTF 125–190 (kcal/kg/day) | RUTF 50–166 (kcal/kg/day) |  | RUTF 1000 kcal/day (2 sachets/day) | RUTF 500 kcal/day (1 sachet/day) |
| *Calculation of dosage* | According to the weight | Fixed amount, regardless of weight or MUAC status | - |  | According to MUAC status and weight | | |  | According to MUAC status | |
| *Frequency* | Weekly | Weekly | No visit |  | Weekly | Weekly | Weekly |  | Weekly | Weekly |
| *Recovery definition* | - MUAC ≥125 mm AND WHZ ≥−2 for 2 consecutive weeks - No edema for 14 days - 4 weeks of participation in the program - Good clinical health (no fever) | | |  | - MUAC ≥125 mm for 2 consecutive weeks - No edema for 2 consecutive weeks - 4 weeks of participation in the program - Good clinical health (no fever) | | |  | - MUAC ≥125 mm for 2 consecutive weeks - No edema for 2 consecutive weeks - 4 weeks of participation in the program - Good clinical health (no fever) | |

ComPAS= Combined protocol for Acute Malnutrition Study ; MAM= Moderate Acute Malnutrition; MUAC= Mid-Upper Arm Circumference; OptiMA= Optimising treatemnt for acute MAlnutrition; RUSF= Ready-to- Use Supplementary Food; RUTF= Ready-to- Use Therapeutic Food ; SAM= Severe Acute Malnutrition; WHZ= Weight-for- Height z-score.

**Supplementary Table 2: RUTF and RUSF ration per week according to Standard protocol of Niger, OptiMA and ComPAS strategies.**

| Standard protocol of Niger | | | | | OPTIMA | | | | | | |  | | ComPAS | | |
| --- | --- | --- | --- | --- | --- | --- | --- | --- | --- | --- | --- | --- | --- | --- | --- | --- |
|  | Edema or MUAC < 115 or WHZ <-3Z |  | MUAC 115-124 or WHZ -3 and -2 AND aged 6-24m |  | | MUAC< 115 mm or Edema |  | MUAC 115-119 mm |  | MUAC 120-124 mm |  | | MUAC<115 mm or Edema | | MUAC 115-124 mm |  |
|  |  |  |  |  |  |  |  |  |  |  |  | |  |  |  |  |
|  |  |  |  |  |  |  |  |  |  |  |  | |  |  |  |  |
| Weight (kg) | RUTF sachets/week |  | RUSF | Weight (kg) | | RUTF sachets/week |  | RUTF sachets/week |  | RUTF sachets/week |  | | RUTF sachets/week | | RUTF sachets/week |  |
| 3.0-3.4 | 8 |  | 7 sachets / week | 3.0-3.4 | | 10 |  | 8 |  | 7 |  | |  | |  |  |
| 3.5 -4.9 | 10 |  |  | 3.5-4.0 | | 11 |  | 8 |  | 7 |  | | 14 sachets / week | |  |  |
|  |  |  |  | 4.1-4.4 | | 12 |  | 9 |  | 7 |  | |  |  | 7sachets / week |  |
|  |  |  |  | 4.5-4.9 | | 13 |  | 10 |  | 7 |  | |  |  |  |  |
| 5.0-6.9 | 15 |  |  | 5.0-5.4 | | 14 |  | 11 |  | 7 |  | |  |  |  |  |
|  |  |  |  | 5.5-5.9 | | 15 |  | 12 |  | 7 |  | |  |  |  |  |
|  |  |  |  | 6.0-6.4 | | 16 |  | 12 |  | 7 |  | |  |  |  |  |
|  |  |  |  | 6.5-6.9 | | 17 |  | 13 |  | 7 |  | |  |  |  |  |
| 7.0-9.9 | 20 |  |  | 7.0-7.4 | | 18 |  | 13 |  | 8 |  | |  |  |  |  |
|  |  |  |  | 7.5-7.9 | | 19 |  | 14 |  | 8 |  | |  |  |  |  |
|  |  |  |  | 8.0-8.4 | | 20 |  | 15 |  | 9 |  | |  |  |  |  |
|  |  |  |  | 8.5-8.9 | | 22 |  | 15 |  | 9 |  | |  |  |  |  |
|  |  |  |  | 9.0-9.4 | | 23 |  | 16 |  | 9 |  | |  |  |  |  |
|  |  |  |  | 9.5-9.9 | | 24 |  | 17 |  | 9 |  | |  |  |  |  |
| 10.0-14.9 | 30 |  |  | 10.0-10.4 | | 25 |  | 18 |  | 10 |  | |  |  |  |  |
|  |  |  |  | 10.5-10.9 | | 26 |  | 19 |  | 10 |  | |  |  |  |  |
|  |  |  |  | 11.0-11.4 | | 27 |  | 20 |  | 10 |  | |  |  |  |  |
|  |  |  |  | 11.5-11.9 | | 29 |  | 21 |  | 10 |  | |  |  |  |  |
|  |  |  |  | 12.0-12.4 | | 30 |  | 22 |  | 11 |  | |  |  |  |  |
|  |  |  |  | 12.5-12.9 | | 31 |  | 22 |  | 11 |  | |  |  |  |  |
|  |  |  |  | 13.0-13.4 | | 33 |  | 23 |  | 12 |  | |  |  |  |  |
|  |  |  |  | 13.5-13.9 | | 34 |  | 24 |  | 12 |  | |  |  |  |  |
|  |  |  |  | 14.0-14.4 | | 35 |  | 25 |  | 12 |  | |  |  |  |  |
|  |  |  |  | 14.5-14.9 | | 36 |  | 26 |  | 13 |  | |  |  |  |  |
| 15.0-19.9 | 35 |  |  | 15.0-15.4 | | 36 |  | 28 |  | 14 |  | |  |  |  |  |
|  |  |  |  | 15.5-15.9 | | 36 |  | 28 |  | 14 |  | |  |  |  |  |
|  |  |  |  | 16.0-16.4 | | 36 |  | 28 |  | 14 |  | |  |  |  |  |
|  |  |  |  | 16.5-16.9 | | 36 |  | 28 |  | 14 |  | |  |  |  |  |
|  |  |  |  | 17.0-17.4 | | 36 |  | 28 |  | 14 |  | |  |  |  |  |
|  |  |  |  | 17.5-17.9 | | 36 |  | 28 |  | 14 |  | |  |  |  |  |
|  |  |  |  | 18.0-18.4 | | 36 |  | 28 |  | 14 |  | |  |  |  |  |
|  |  |  |  | 18.5-18.9 | | 36 |  | 28 |  | 14 |  | |  |  |  |  |
|  |  |  |  | 19.0-19.4 | | 36 |  | 28 |  | 14 |  | |  | |  |  |
|  |  |  |  | 19.5-19.9 | | 36 |  | 28 |  | 14 |  | |  | |  |  |

MUAC= Mid-Upper Arm Circumference; OptiMA= Optimising treatemnt for acute MAlnutrition; RUSF= Ready-to- Use Supplementary Food; RUTF= Ready-to- Use Therapeutic Food ; WHZ= Weight-for- Height z-score.

**Supplementary Table 3: Baseline characteristics in children admitted with SAM according to WHO definition (MUAC<115mm or WHZ<-3 or edema)**

|  | Children with MUAC<115mm or WHZ<-3 or edema | | |
| --- | --- | --- | --- |
| Variable | Standard, N = 299 | OptiMA, N = 297 | ComPAS, N = 296 |
| Sociodemographic characteristics |  |  |  |
| Sex |  |  |  |
| Female | 129 (43·1) | 157 (52·9) | 155 (52·4) |
| Male | 170 (56·9) | 140 (47·1) | 141 (47·6) |
| Age, months | 17 (11, 23) | 16 (10, 22) | 16 (10, 23) |
| Health center |  |  |  |
| Diney | 38 (12·7) | 37 (12·5) | 38 (12·8) |
| Droum | 131 (43·8) | 129 (43·4) | 125 (42·2) |
| Gada | 52 (17·4) | 52 (17·5) | 54 (18·2) |
| Gafati | 78 (26·1) | 79 (26·6) | 79 (26·7) |
| Currently breastfed | 156 (52·2) | 171 (57·6) | 165 (55·7) |
| Mother alive | 298 (99·7) | 297 (100·0) | 291 (98·3) |
| Mother as caretaker | 275 (92·0) | 286 (96·3) | 278 (93·9) |
| Caretaker was illiterate | 286 (95·7) | 282 (94·9) | 279 (94·3) |
| First child born | 15 (5·0) | 7 (2·4) | 14 (4·7) |
| Distance from health center, km |  |  |  |
| >=10 | 87 (29·1) | 78 (26·3) | 89 (30·1) |
| Number of siblings | 4·0 (2·0, 6·0) | 4·0 (2·0, 6·0) | 4·0 (2·0, 6·0) |
| Number of people living in the household | 7·0 (4·0, 10·0) | 6·0 (4·0, 9·0) | 7·0 (5·0, 10·0) |
| Number of children participating in OTP in the household |  |  |  |
| >= 1child | 28 (9·4) | 21 (7·1) | 33 (11·1) |
| Anthropometric characteristics |  |  |  |
| MUAC, mm | 112·0 (107·5, 115·0) | 112·0 (107·0, 115·0) | 112·0 (108·0, 115·0) |
| <115 or edema | 218 (72·9) | 215 (72·4) | 218 (73·6) |
| Nutritional edema |  |  |  |
| + | 9 (3·0) | 8 (2·7) | 6 (2·0) |
| ++ | 3 (1·0) | 2 (0·7) | 2 (0·7) |
| Weight, kg | 6·2 (5·5, 6·8) | 6·1 (5·4, 6·8) | 6·1 (5·5, 6·9) |
| Height, cm | 70·0 (65·5, 73·2) | 69·5 (65·0, 73·3) | 69·5 (65·5, 73·6) |
| WHZ | -3·3 (-3·8, -3·1) | -3·3 (-3·8, -3·0) | -3·3 (-3·7, -2·9) |
| >=-3/<-2 | 67 (22·4) | 81 (27·3) | 75 (25·3) |
| <-3 | 221 (73·9) | 206 (69·4) | 193 (65·2) |
| HAZ | -3·7 (-4·7, -2·6) | -3·6 (-4·6, -2·7) | -3·6 (-4·4, -2·6) |
| >=-3/<-2 | 59 (19·7) | 63 (21·2) | 56 (18·9) |
| <-3 | 197 (65·9) | 195 (65·7) | 198 (66·9) |
| WAZ | -4·3 (-5·0, -3·7) | -4·2 (-4·8, -3·8) | -4·2 (-4·8, -3·7) |
| >=-3/<-2 | 17 (5·7) | 20 (6·7) | 15 (5·1) |
| <-3 | 278 (93·0) | 275 (92·6) | 278 (93·9) |
| Clinical characteristics |  |  |  |
| Temperature axillary |  |  |  |
| >=37.5 | 59 (19·7) | 59 (19·9) | 62 (20·9) |
| Cardiac Frequency (bpm) |  |  |  |
| >=130 | 98 (32·8) | 88 (29·6) | 103 (34·8) |
| Respiratory Frequency (bpm) |  |  |  |
| >=40 | 50 (16·7) | 51 (17·2) | 28 (9·5) |
| Oxygen saturation (%) |  |  |  |
| <92 | 0 (0·0) | 0 (0·0) | 1 (0·3) |
| Malaria rapid antigen test realized |  |  |  |
| Positive | 70 (23·4) | 63 (21·2) | 73 (24·7) |
| Amoxicillin received | 299 (100·0) | 296 (100·0) | 296 (100·0) |
| Missing data | 0 | 1 | 0 |
| Health problem at admission | 196 (65·6) | 197 (66·3) | 198 (66·9) |
| Diarrhea | 63 (21·1) | 68 (22·9) | 73 (24·7) |
| Respiratory infection | 57 (19·1) | 41 (13·8) | 35 (11·8) |
| Nutritional supplementation |  |  |  |
| Eligible for supplementation | 299 (100·0) | 297 (100·0) | 296 (100·0) |
| RUSF | 2 (0·7) | 0 (0·0) | 0 (0·0) |
| RUTF | 297 (99·3) | 297 (100·0) | 296 (100·0) |

Data are n (%) or median (IQR). Severe acute malnutrition (SAM) is defined as MUAC<115 mm, or WHZ score <–3 or edema. Bpm= beats per min. HAZ=height-for-age Z. MUAC=mid-upper-arm circumference. OTP=outpatient therapeutic program. RUSF=ready-to-use supplementary food. RUTF=ready-to-use therapeutic food. WAZ=weight-for-age Z. WHZ=weight for-height Z.

**Supplementary Table 4: Recovery outcome throughout the 6 months follow-up in children admitted with SAM according to WHO definition (MUAC<115mm or WHZ<-3 or edema)**

|  | Intention-to-treat analysis | | | Per protocol analysis‡ | | |
| --- | --- | --- | --- | --- | --- | --- |
| Variable | Standard, N = 299 | OptiMA, N = 297 | ComPAS, N = 296 | Standard, N = 249 | OptiMA, N = 244 | ComPAS, N = 243 |
| Nutritional recovery* | 240 (80·3) | 203 (68·4) | 220 (74·3) | 206 (82·7) | 179 (73·4) | 192 (79·0) |
| No nutritional recovery |  |  |  |  |  |  |
| Death | 10 (3·3) | 9 (3·0) | 5 (1·7) | 10 (4·0) | 9 (3·7) | 5 (2·1) |
| Discontinued trial† | 3 (1·0) | 10 (3·4) | 7 (2·4) |  |  |  |
| MUAC>=125mm and no edema one week only | 9 (3·0) | 28 (9·4) | 22 (7·4) | 8 (3·2) | 19 (7·8) | 12 (4·9) |
| Never reached MUAC>=125mm and no edema | 32 (10·7) | 41 (13·8) | 35 (11·8) | 25 (10·0) | 32 (13·1) | 31 (12·8) |
| RUTF less than 4 visits | 5 (1·7) | 6 (2·0) | 7 (2·4) | 0 (0·0) | 5 (2·0) | 3 (1·2) |

Data are n (%), unless stated otherwise. *Assessed over the 6 months follow-up trial period: after a 4-week minimum duration of supplementation, an axillary temperature less than 37.5 ◦C, Mid-upper-arm circumference ≥125 mm and no bilateral nutritional edema for two consecutive weeks. ‡Per-protocol definition: minimum prescription of 4 weeks of supplementation, children received at least 90% of the total amount of supplementation they were supposed to receive as per protocol and a maximum interval of 8 weeks between any two visits in the 6-month follow-up. †Family moved out of study area or children were lost to follow-up. MUAC=mid-upper-arm circumference. RUTF= ready-to-use therapeutic food.

**Supplementary Table 5: Follow-up characteristics during the 6-month trial period in children admitted with SAM according to WHO definition (MUAC<115mm or WHZ<-3 or edema) (intention-to-treat analysis)**

|  | Children with MUAC<115mm or WHZ<-3 or edema (+, ++) | | | | |  |
| --- | --- | --- | --- | --- | --- | --- |
| Variable | Standard, N = 299 | OptiMA, N = 297 | p-value | ComPAS, N = 296 | p-value | |
| Completed 6 month follow-up | 267 (89·3) | 255 (85·9) | 0·48 | 266 (89·9) | 0·41 | |
| Program attendance |  |  |  |  |  | |
| Follow-up visits at the trial center, visits | 11·0 (7·0, 16·0) | 11·0 (7·0, 16·0) | 0·76 | 11·0 (7·0, 15·2) | 0·41 | |
| Follow-up visits at home, visits | 3·0 (2·0, 4·0) | 3·0 (1·0, 4·0) | 0·18 | 3·0 (2·0, 4·0) | 0·90 | |
| At least one missed visit at health center | 122 (40·8) | 157 (52·9) | 0·003 | 156 (52·7) | 0·004 | |
| At least one missed visit at home | 96 (32·1) | 110 (37·0) | 0·21 | 104 (35·1) | 0·43 | |
| Comorbidities |  |  |  |  |  | |
| At least one health problem during the 6 month follow-up | 291 (97·3) | 294 (99·0) | 0·13 | 292 (98·6) | 0·25 | |
| At least one RDT malaria positive during the 6 month follow-up | 196 (65·6) | 219 (73·7) | 0·030 | 207 (69·9) | 0·25 | |
| At least one IRA episode during the 6 month follow-up | 175 (58·5) | 167 (56·2) | 0·57 | 154 (52·0) | 0·11 | |
| At least one diarrhea episode during the 6 month follow-up | 176 (58·9) | 168 (56·6) | 0·57 | 175 (59·1) | 0·95 | |
| At least one anemia episode during the 6 month follow-up | 9 (3·0) | 9 (3·0) | 0·99 | 14 (4·7) | 0·28 | |
| Hospitalization |  |  |  |  |  | |
| Children with at least one follow-up visit with indication for reference to hospital | 183 (61·2) | 189 (63·6) | 0·54 | 181 (61·1) | 0·99 | |
| Main reason for reference to hospital: stagnant or weight loss | 161 (53·8) | 170 (57·2) | 0·40 | 165 (55·7) | 0·64 | |
| Children hospitalized at least once | 89 (29·8) | 89 (30·0) | 0·96 | 72 (24·3) | 0·14 | |
| Main diagnosis - Malaria | 46 (51·7) | 48 (53·9) | 0·76 | 49 (68·1) | 0·036 | |
| MUAC at hospitalization, mm | 115·0 (108·0, 120·0) | 113·0 (108·0, 118·0) | 0·43 | 114·0 (110·0, 116·2) | >0·99 | |
| Nutritional edema at hospitalization | 3 (3·4) | 4 (4·5) | >0·99 | 6 (8·3) | 0·30 | |
| Length of therapeutic milk F-75 received | 2·0 (2·0, 3·0) | 2·0 (2·0, 3·0) | 0·43 | 2·0 (1·5, 3·0) | 0·84 | |
| Length of therapeutic milk F-100 received | 1·0 (0·0, 1·0) | 1·0 (1·0, 2·0) | 0·10 | 1·0 (0·8, 2·2) | 0·22 | |
| Length of RUTF received, days | 2·0 (2·0, 3·0) | 2·0 (2·0, 3·0) | 0·23 | 2·0 (2·0, 3·0) | 0·90 | |
| MUAC at the end of hospitalization, mm‡ | 116·0 (110·0, 121·0) | 115·0 (110·0, 120·0) | 0·51 | 115·0 (112·0, 120·0) | 0·88 | |
| Length of hospitalization, days‡ | 4·0 (3·0, 6·0) | 5·0 (3·0, 6·0) | 0·54 | 4·5 (3·0, 6·0) | 0·38 | |

Data are median (IQR) or n (%). ‡Calculated at first hospitalization. HAZ=height-for-age Z. MUAC=mid-upper-arm circumference. RUSF=ready-to-use supplementary food. RUTF=ready-to-use therapeutic food. WAZ=weight-for-age Z. WHZ=weight for-height Z.

**Supplementary Table 6: Nutritional and anthropometric characteristics over 6 months post-inclusion in children admitted with SAM according to WHO definition (MUAC<115mm or WHZ<-3 or edema) (intention-to-treat analysis)**

|  | Children with MUAC<115mm or WHZ<-3 or edema (+, ++) | | | | |  |
| --- | --- | --- | --- | --- | --- | --- |
| Variable | Standard, N = 299 | OptiMA, N = 297 | p-value | ComPAS, N = 296 | p-value | |
| Anthropometric characteristics at 6 month |  |  |  |  |  | |
| MUAC, mm | 130·0 (125·0, 135·0) | 127·0 (122·0, 133·0) | 0·002 | 130·0 (124·0, 134·0) | 0·22 | |
| MUAC <115 mm or edema | 22 (7·4) | 17 (5·7) |  | 17 (5·7) | 0·16 | |
| Weight, kg | 8·1 (7·3, 9·0) | 7·8 (7·0, 8·9) | 0·024 | 8·1 (7·4, 8·9) | 0·81 | |
| Height, cm | 72·0 (68·3, 76·0) | 72·0 (67·7, 76·0) | 0·48 | 72·3 (68·0, 76·5) | 0·82 | |
| WHZ | -0·8 (-1·6, -0·1) | -1·0 (-1·9, -0·3) | 0·034 | -0·8 (-1·5, -0·2) | 0·81 | |
| WHZ <-3 | 17 (5·7) | 17 (5·7) | 0·051 | 14 (4·7) | 0·37 | |
| HAZ* | -4·3 (-5·1, -3·4) | -4·2 (-5·1, -3·4) | 0·51 | -4·1 (-4·9, -3·5) | 0·36 | |
| HAZ <-3 * | 250 (83·6) | 251 (84·5) | 0·84 | 253 (85·5) | 0·81 | |
| WAZ* | -3·0 (-3·7, -2·2) | -3·1 (-3·9, -2·3) | 0·31 | -3·0 (-3·6, -2·3) | 0·65 | |
| WAZ <-3* | 147 (49·2) | 159 (53·5) | 0·57 | 142 (48·0) | 0·12 | |
| Change in anthropometric parameters between inclusion and 6 months | | | | | | |
| Duration in the trial, week | 26·0 (25·6, 26·3) | 26·0 (25·4, 26·3) | 0·12 | 26·0 (25·6, 26·3) | 0·57 | |
| MUAC gain, mm | 17 (12, 24) | 16 (10, 23) | 0·038 | 17 (11, 23) | 0·35 | |
| WEIGHT gain, g | 2 000 (1 400, 2 600) | 1 800 (1 200, 2 400) | 0·010 | 1 900 (1 400, 2 400) | 0·22 | |
| Height gain, cm | 2·5 (1·5, 3·5) | 2·5 (1·5, 3·5) | 0·95 | 2·5 (1·5, 3·5) | 0·63 | |
| Daily weight gain, g/kg per day | 1·9 (1·4, 2·5) | 1·8 (1·2, 2·5) | 0·080 | 1·7 (1·3, 2·3) | 0·041 | |
| Weekly MUAC gain, mm per week | 0·7 (0·5, 1·0) | 0·7 (0·4, 1·0) | 0·071 | 0·7 (0·5, 0·9) | 0·12 | |
| Nutritional improvement |  |  |  |  |  | |
| Children who recovered during the 6 month follow-up† | 240 (80·3) | 203 (68·4) | <0·001 | 220 (74·3) | 0·084 | |
| Time to reach recovery† by 6 months, weeks | 9·0 (5·0, 13·2) | 9·0 (6·0, 16·0) | 0·17 | 9·0 (6·0, 16·0) | 0·10 | |
| Children who recovered† by 12 weeks | 169 (56·5) | 130 (43·8) | 0·002 | 137 (46·3) | 0·012 | |
| Children who recovered† by 16 weeks | 202 (67·6) | 153 (51·5) | <0·001 | 166 (56·1) | 0·004 | |
| Nutritional recovery¶ one week only | 252 (84·3) | 240 (80·8) | 0·26 | 249 (84·1) | 0·96 | |
| Achieving primary outcome ^£^ | 165 (55·2) | 134 (45·1) | 0·014 | 157 (53·0) | 0·60 | |
| Nutritional treatment distributed |  |  |  |  |  | |
| Children non eligible to supplementation | 0 (0·0) | 0 (0·0) | - | 0 (0·0) | - | |
| Median amount of RUTF/RUSF received during the 6 months follow-up, sachet | 180·0 (124·5, 250·0) | 122·0 (75·0, 185·0) | <0·001 | 91·0 (56·0, 140·0) | <0·001 | |
| Median length of RUTF/RUSF treatment during the 6 months follow-up, days | 77·0 (49·0, 105·0) | 77·0 (49·0, 112·0) | 0·56 | 77·0 (49·0, 105·0) | 0·85 | |
| Median amount of RUTF/RUSF received during the initial episode, sachet | 165·0 (115·0, 225·0) | 108·0 (65·0, 181·0) | <0·001 | 77·0 (56·0, 133·0) | <0·001 | |
| Median length of RUTF/RUSF treatment during the initial episode, days | 70·0 (49·0, 98·0) | 70·0 (49·0, 105·0) | 0·31 | 63·0 (42·0, 98·0) | 0·77 | |
| Energy received during the initial episode in kcal/kg/d | 186·7 (176·9, 197·5) | 123·3 (104·8, 148·6) | <0·001 | 97·0 (80·5, 121·5) | <0·001 | |

Data are median (IQR) or n HAZ=height-for-age Z. MUAC=mid-upper-arm circumference. RUSF=ready-to-use supplementary food. RUTF=ready-to-use therapeutic food. WAZ=weight-for-age Z. WHZ=weight for-height Z. *1 children aged more than 59 months were excluded from this analysis ; † Assessed after a 4-week minimum duration of supplementation, an axillary temperature less than 37.5 ◦C, Mid-upper-arm circumference ≥125 mm and no bilateral nutritional edema for two consecutive weeks. ^¶^ Assessed over the 6 months follow-up trial period, defined as reaching a mid-upper-arm circumference ≥125 mm and no bilateral nutritional edema ^£^Assessed 6 months after inclusion: child alive, not acutely malnourished (mid-upper-arm circumference ≥125 mm, no bilateral nutritional edema, and weight-for-height Z score ≥−3), and not having experienced any new episode of acute malnutrition until at least a 6-month post-enrollment follow-up.

**Supplementary Table 7: Characteristics of deceased children during the 6-month period**

| Variable | Standard  N = 23 | OptiMA  N = 19 | ComPAS  N = 11 |
| --- | --- | --- | --- |
| Characteristics at admission |  |  |  |
| Sex |  |  |  |
| Female | 12 (52·2) | 12 (63·2) | 5 (45·5) |
| Male | 11 (47·8) | 7 (36·8) | 6 (54·5) |
| Age, months | 16·0 (9·5, 25·5) | 16·0 (12·5, 22·5) | 18·0 (10·0, 23·0) |
| Health center |  |  |  |
| Diney | 2 (8·7) | 2 (10·5) | 1 (9·1) |
| Droum | 11 (47·8) | 6 (31·6) | 7 (63·6) |
| Gada | 3 (13·0) | 5 (26·3) | 1 (9·1) |
| Gafati | 7 (30·4) | 6 (31·6) | 2 (18·2) |
| MUAC, mm | 116·0 (107·5, 120·0) | 112·0 (109·5, 118·0) | 115·0 (106·5, 119·5) |
| MUAC <115mm or edema | 11 (47·8) | 11 (57·9) | 5 (45·5) |
| Nutritional edema |  |  |  |
| + | 1 (4·3) | 0 (0·0) | 0 (0·0) |
| ++ | 1 (4·3) | 0 (0·0) | 0 (0·0) |
| Weight, kg | 6·3 (5·8, 6·9) | 6·4 (5·5, 7·0) | 6·4 (5·8, 6·9) |
| WHZ | -2·9 (-3·7, -2·3) | -3·6 (-3·9, -3·0) | -3·2 (-3·6, -2·9) |
| HAZ | -3·7 (-4·7, -2·0) | -3·6 (-5·0, -2·2) | -4·2 (-4·5, -2·7) |
| WAZ | -4·0 (-5·0, -3·3) | -4·3 (-5·1, -3·3) | -4·5 (-4·8, -3·5) |
| Positive malaria rapid antigen test | 2 (8·7) | 2 (10·5) | 1 (9·1) |
| Health problem | 12 (52·2) | 14 (73·7) | 8 (72·7) |
| Supplementation initiated |  |  |  |
| Not eligible for supplementation | 3 (13·0) | 0 (0·0) | 0 (0·0) |
| RUSF | 8 (34·8) | 0 (0·0) | 0 (0·0) |
| RUTF | 12 (52·2) | 19 (100·0) | 11 (100·0) |
| Follow-up duration |  |  |  |
| Number of visits at the trial center, visits | 6·0 (3·0, 8·5) | 8·0 (5·0, 12·0) | 6·0 (4·5, 8·5) |
| Follow-up duration, days | 56·0 (32·0, 86·5) | 73·0 (56·0, 126·5) | 56·0 (29·5, 121·0) |
| Hospitalization |  |  |  |
| Children with at least one follow-up visit with indication for reference to hospital | 11 (47·8) | 11 (57·9) | 7 (63·6) |
| Children hospitalized at least once | 8 (34·8) | 5 (26·3) | 7 (63·6) |
| Anthropometric criteria at the latest visit prior to death | | |  |
| MUAC (mm) | 116·0 (108·5, 125·0) | 125·0 (116·5, 126·5) | 115·0 (108·5, 120·0) |
| Weight (kg) | 6·6 (6·0, 7·8) | 7·1 (6·1, 8·0) | 6·4 (5·6, 7·3) |
| Main diagnostic of death |  |  |  |
| Severe malaria | 12 (52·2) | 11 (57·9) | 5 (45·5) |
| Gastroenteritis | 4 (17·4) | 2 (10·5) | 4 (36·4) |

Data are median (IQR) or n (%). HAZ=height-for-age Z. MUAC=mid-upper-arm circumference. RUSF=ready-to-use supplementary food. RUTF=ready-to-use therapeutic food. WAZ=weight-for-age Z. WHZ=weight for-height Z.

#### Supplementary Table 8: Recovery outcome throughout the 6 months follow-up in children admitted with MUAC<115mm or edema and who reached a MUAC>125mm and no edema one week (intention-to-treat analysis)

|  | Intention to treat analysis | | | Per protocol analysis‡ | | |
| --- | --- | --- | --- | --- | --- | --- |
| Variable | Standard, N= 382 | OptiMA, N=379 | ComPAS, N=379 | Standard, N=323 | OptiMA, N=293 | ComPAS, N=298 |
| Reaching a MUAC>125mm and no edema | 314 (82·2) | 300 (79·2) | 304 (80·2) | 276 (85·4) | 250 (85·3) | 254 (85·2) |
| Causes of not reaching a MUAC>125mm and no edema |  |  |  |  |  |  |
| Death | 11 (2·9) | 6 (1·6) | 5 (1·3) | 11 (3·4) | 6 (2·0) | 5 (1·7) |
| Discontinued trial† | 11 (2·9) | 22 (5·8) | 23 (6·1) | 0 (0·0) | 0 (0·0) | 0 (0·0) |
| RUTF less than 4 visits | 1 (0·3) | 1 (0·3) | 1 (0·3) | 0 (0·0) | 0 (0·0) | 0 (0·0) |
| Never reached MUAC>125mm and no edema one week | 45 (11·8) | 50 (13·2) | 46 (12·1) | 36 (11·1) | 37 (12·6) | 39 (13·1) |

Data are n (%), unless stated otherwise. *Assessed over the 6 months follow-up trial period. ‡Per-protocol definition: minimum prescription of 4 weeks of supplementation, children received at least 90% of the total amount of supplementation they were supposed to receive as per protocol and a maximum interval of 8 weeks between any two visits in the 6-month follow-up. †Family moved out of study area or children were lost to follow-up. MUAC=mid-upper-arm circumference. RUTF= ready-to-use therapeutic food.

**Supplementary Table 9: Follow-up characteristics in children admitted with MUAC<115mm or edema and who reached a MUAC>125mm and no edema one week (intention-to-treat analysis)**

| Variable |  |  |  |  |  |
| --- | --- | --- | --- | --- | --- |
| Group | Standard, N = 314 | OptiMA, N = 300 | p-value | ComPAS, N = 304 | p-value |
| Program attendance |  |  |  |  |  |
| Completeness of follow-up |  |  | 0·092 |  | 0·32 |
| Completed 6 month follow-up | 297 (94·6) | 268 (89·3) |  | 284 (93·4) |  |
| Death | 3 (1·0) | 7 (2·3) |  | 1 (0·3) |  |
| Family moved out of study area | 1 (0·3) | 4 (1·3) |  | 0 (0·0) |  |
| Lost to follow-up | 13 (4·1) | 21 (7·0) |  | 19 (6·2) |  |
| Follow-up duration, months | 6·0 (5·9, 6·0) | 6·0 (5·9, 6·0) | 0·42 | 6·0 (5·9, 6·0) | 0·42 |
| Follow-up visits at the trial center, visits | 11·0 (8·0, 15·0) | 10·5 (7·0, 15·0) | 0·28 | 10·0 (7·0, 14·0) | 0·054 |
| Follow-up visits at home, visits | 3·0 (2·0, 4·0) | 3·0 (2·0, 4·0) | 0·39 | 3·0 (2·0, 4·0) | 0·87 |
| At least one missed visit at health center | 121 (38·5) | 165 (55·0) | <0·001 | 171 (56·2) | <0·001 |
| At least one missed visit at home | 98 (31·2) | 116 (38·7) | 0·053 | 106 (34·9) | 0·33 |
| Hospitalization during the 6 month follow-up | | | | | |
| Children with at least one follow-up visit with indication for reference to hospital | 184 (58·6) | 182 (60·7) | 0·60 | 177 (58·2) | 0·92 |
| Main reason for reference to hospital: stagnant or weight loss | 161 (51·3) | 165 (55·0) | 0·36 | 160 (52·6) | 0·74 |
| Children hospitalized at least once | 65 (20·7) | 68 (22·7) | 0·55 | 56 (18·4) | 0·48 |
| Main diagnosis - Malaria | 35 (53·8) | 43 (63·2) | 0·27 | 40 (71·4) | 0·047 |
| MUAC, mm | 113·0 (108·0, 118·0) | 113·5 (106·0, 117·0) | 0·71 | 114·5 (110·0, 118·0) | 0·46 |
| MUAC cateory |  |  | 0·95 |  | 0·67 |
| <115 | 35 (53·8) | 37 (54·4) |  | 28 (50·0) |  |
| >=115 | 30 (46·2) | 31 (45·6) |  | 28 (50·0) |  |
| Nutritonal edema | 1 (1·5) | 1 (1·5) | >0·99 | 5 (8·9) | 0·095 |
| Length of therapeutic milk F-75 received | 2·0 (1·0, 2·0) | 2·0 (1·5, 3·0) | 0·31 | 2·0 (1·0, 3·0) | 0·78 |
| Length of therapeutic milk F-100 received | 0·5 (0·0, 1·0) | 2·0 (1·0, 2·0) | 0·051 | 1·5 (1·0, 2·8) | 0·069 |
| Length of RUTF received, days | 2·0 (2·0, 3·0) | 2·0 (2·0, 3·0) | 0·14 | 2·0 (2·0, 3·0) | 0·85 |
| MUAC at the end of hospitalization, mm | 114·0 (110·0, 119·5) | 115·0 (109·2, 119·8) | 0·79 | 115·5 (112·0, 120·8) | 0·15 |
| Length of hospitalization, days | 4·0 (3·0, 5·0) | 5·0 (4·0, 6·0) | 0·10 | 5·0 (3·0, 6·0) | 0·33 |

Data are median (IQR) or n (%). MUAC=mid-upper-arm circumference. RUTF= ready-to-use therapeutic food.

**Supplementary Figures**

**Supplementary Figure 1: Intention-to-treat (ITT) and Per-protocol (PP) non-inferiority analysis of nutritional recovery outcome in children admitted with SAM according to WHO definition (MUAC<115mm or WHZ<-3 or edema)** ITT = Intention-to-treat analysis ; MUAC = mid-upper arm circumference ; PP = Per protocol analysis.

**Supplementary Figure 2: Modelled adjusted weekly means of weight, MUAC and height cumulated gain through over 6-month by randomization groups (intention-to-treat).** MUAC = mid-upper-arm circumference. WHZ = weight for-height z-score.

**Supplementary Figure 3: Sensibility non-inferiority analysis of children who reach a MUAC>125mm one week only without edema over the 6-months follow-up trial period.** ITT = Intention-to-treat analysis ; MUAC = mid-upper arm circumference ; PP = Per protocol analysis.
